# Impact of the Chemical Structure of Photoreactive Urethane (Meth)Acrylates with Various (Meth)Acrylate Groups and Built-In Diels–Alder Reaction Adducts on the UV-Curing Process and Self-Healing Properties

**DOI:** 10.3390/polym15040924

**Published:** 2023-02-12

**Authors:** Paulina Bednarczyk, Karolina Mozelewska, Joanna Klebeko, Joanna Rokicka, Paula Ossowicz-Rupniewska

**Affiliations:** Department of Chemical Organic Technology and Polymeric Materials, Faculty of Chemical Technology and Engineering, West Pomeranian University of Technology in Szczecin, Piastów Ave. 42, 71-065 Szczecin, Poland

**Keywords:** self-healing polymers, photopolymerization, urethane acrylate oligomers, smart polymers, coatings, photocuring, Diels–Alder reaction

## Abstract

A series of UV-curable urethane (meth)acrylates were obtained by copolymerization of the Diels–Alder adduct (HODA), isophorone diisocyanate, PEG1000, and various hydroxy (meth)acrylates. The aim of the present work was to determine the influence of the chemical structure of the introduced (meth)acrylic groups, i.e., hydroxyethyl acrylate, hydroxypropyl acrylate, hydroxyethyl methacrylate, and hydroxypropyl methacrylate, on the UV-curing process and self-healing properties of cured coatings. The chemical structure of prepolymers was characterized by FTIR and NMR spectroscopy, whereas the UV-curing process was monitored in real time using FTIR and photo-DSC. In turn, the self-healing properties were characterized in relation to the thermally reversible mechanism, which was tested using the following methods: an FTIR spectroscope equipped with a heating attachment; DSC and TG apparatus; and an optical microscope equipped with a stage with programmable heating. The result of comprehensive research on the self-healing of photocurable coatings in the context of the presence of various photoreactive groups and the course of the curing process allows one to control the self-healing process by reducing the effective healing temperature. The self-healing properties, taken together with the fast UV curing of the coatings and excellent properties of cured coatings, make the material attractive for a variety of applications, in particular in cases where coatings are not repaired, e.g., for economic reasons or when it is not possible, such as in flexible electronic screens, car paint film, and aircraft interior finishes.

## 1. Introduction

There is a growing interest in self-healing polymeric materials and photocurable coatings. For this purpose, a reversible Diels–Alder reaction was chosen. This reaction is one of the most effective and reliable strategies for producing cyclic hydrocarbons, for which explorers Otto Diels and Kurt Alder won the Nobel Prize in 1950 [1]. A key element in the synthesis of this type of polymeric material is the incorporation of Diels–Alder adducts into the polymer structure and the use of appropriate adducts, which enable the coordinated cycloaddition of the substituted alkene to the conjugated diene and the possibility of self-repair of the polymer [2,3,4,5]. Self-healing materials respond to structural damage such as cracks, cavities, and dents. A material self-healing mechanism is analogous to that in nature (e.g., skin healing and broken bones fused) and is based on an immediate response to damage without (or with minimal) human intervention. The speed of reaction with minimal participation from external factors is also important [6,7,8,9,10].

So far, research has been conducted on functional materials based on thermally reversible Diels–Alder reactions. Specifically, the Diels–Alder reaction is based on the furan-maleimide structure, which combines maleimide’s excellent effect with the possibility of obtaining furan derivatives from renewable sources. Therefore, the concept is widely used in the fields of recyclable materials, intelligent composite materials, and smart coatings. There is a lack of information about the preparation of monomers or resins containing Diels–Alder adducts in which UV curing is used. Vilela et al. prepared monomers based on triglycerides with incorporated furan moieties and then polymerized these monomers using Diels–Alder polycondensation [11]. Additionally, Huang et al. used the Diels–Alder reaction to join maleic anhydride to tung oil resin, which was then changed to make a UV-curable resin [12].

There are many reports on self-healing polyurethanes with a built-in Diels–Alder adduct. Fang et al. reported a self-healing polyurethane based on the reversible Diels–Alder and retro-Diels–Alder reactions using a diol obtained from *N*-(2-hydroxyethyl)maleimide and furfuryl alcohol. [13]. On the other hand, self-healing polyurethanes based on the Diels–Alder reaction between furan and maleimide groups, which use the shape memory effect to self-repair, were described by Heo and Sodano [14]. It is also known that Diels–Alder adduct-1-(hydroxymethyl)-10-oxatricycle [5.2.1.02,6] dec-8-ene-3,5-dione-2-aminoethanol is used in the preparation of polyurethane based on *N*,*N*,*N′*,*N′*-tetrakis(2-hydroxypropyl)ethylenediamine and hexamethylene diisocyanate [15]. Water-soluble polyurethanes with Diels–Alder groups capable of self-repairing were developed by the Irusta group using one-pot reactions based on isophorone diisocyanate, 2-bis(hydroxymethyl)propionic acid, triethanolamine (in the first stage for prepolymer synthesis), and 1,4-butanediol (in the extension stage) chain [16]. Polyurethanes based on isophorone diisocyanate, polylactic acid, 2-hydroxypropyl acrylate, and a Diels–Alder adduct are also known [17]. Copolymers based on bisphenol A ethoxylate diacrylate and tricyclo [5.2.1.02.6] decanedimethanol diacrylate are other UV-curable acrylate copolymers containing Diels–Alder adducts [18]. From the publication of Irust and coresearchers, known polyurethanes based on isophorone diisocyanate and thermally crosslinked polypropylene glycol are involved in the reversible Diels–Alder reaction [19]. Other self-healing polymeric materials were obtained by Fortunato et al. by reacting a series of linear furfuryl methacrylate copolymers with aliphatic bismaleimides in a Diels–Alder reaction between furan and maleimide groups [20]. 

A polyurethane resin containing acrylate and a Diels–Alder structure with the potential to self-repair under the influence of temperature and be cured by UV radiation are described by Ke et al. To introduce the furan-maleimide structure, *N*-hydroxyethyl maleimide with hydroxyl and maleimide groups was reacted with furfuryl alcohol to produce a diol. This diol was then mixed with isophorone diisocyanate (IPDI), polyethylene glycol (PEG1000) as a chain extender, and hydroxypropyl acrylate (HPA) to make a UV-curable polyurethane [21].

This article presents methods for the preparation of new urethane (meth)acrylate resins with a built-in Diels–Alder reaction adduct based on 1-(hydroxymethyl)-10-oxatricyclo [5.2.1.02.6]dec-8-ene-3,5-dione-2-aminoethanol, isophorone diisocyanate, polyethylene glycol, and corresponding acrylate and methacrylate as well as both 2-hydroxyethyl and 2-hydroxypropyl, which are new polymeric materials. These new self-healing polymeric materials can be used in the production of automobile varnishes and other plastic elements used in the automotive industry. The aim of this study is to compare the impact of the modification of polymeric materials and UV-curable coatings based on urethane acrylates with built-in Diels–Alder reaction adducts on their self-healing properties. For this purpose, various acrylates and methacrylates were used in the synthesis. The obtained resins were characterized in terms of the influence of the chemical structure on the photocuring process and the self-healing of the cured coatings.

## 2. Materials and Methods

### 2.1. Materials

All reagents were commercially available materials and were used without further purification. Maleic anhydride (≥98%) and dibutyltin dilaurate (95%) were provided by Alfa Aesar (Haverhill, MA, USA). Furan (≥99%) was obtained from Fluka. Hydroquinone (≥99%), 2-hydroxypropyl acrylate (HPA) (mixture of the isomers, 95%), 2-hydroxyethyl methacrylate (HEMA) (97%), hexamethylene diisocyanate (for synthesis), polyethylene glycol PEG 1000 (for synthesis), and 2-hydroxypropyl methacrylate (HPMA) (mixture of hydroxypropyl and 2-hydroxypropyl methacrylates, ≥97%) were purchased from Sigma-Aldrich (Steinheim am Albuch, Germany). Furfuryl alcohol (98%) and isophorone diisocyanate (98%) were provided by Acros Organics (Geel, Belgium). 2-hydroxyethyl acrylate (HEA) was kindly donated by Cognis Performance Chemicals (Hythe, UK). Chempur (Piekary Śląskie, Poland) provided analytical-grade ethanolamine and triethanolamine. Diethyl ether (99.5%) and anhydrous ethanol p.a. (99.8%) were provided by Avantor Performance Materials Poland S.A. (POCH, Gliwice, Poland). Acetone, isopropanol, xylene, toluene, and dichloromethane of high purity were purchased from StanLab (Lublin, Poland).

### 2.2. Synthesis of UV-Curable UA Resins

Using the previously described and modified four-step method [21,22,23,24], we synthesized the Diels–Alder adduct as 1-(hydroxymethyl)-10-oxatricyclo[5.2.1.02.6]dec-8-ene-3,5-dione-2-aminoethanol (HODA; Figure S1—Steps 1–4).

Step 1: 4,10-dioxatricyclo [5.2.1.02,6] dec-8-ene-3,5-dione (DCD) was obtained by reacting maleic anhydride (BM) with furan (FU) in an acetone medium. The reaction was carried out at room temperature under constant stirring under argon for 24–48 h. The product was separated from the reaction mixture by filtration under reduced pressure, and then the obtained white powder was washed with diethyl ether and dried in a vacuum oven. The yield of this step ranged from 84 to 90%.

Step 2: 4-(2-hydroxyethyl)-10-oxa-4-azatricyclo[5.2.1.02.6]dec-8-ene-3,5-dione (HAD) was obtained by the reaction of 4,10-dioxatricyclo[5.2.1.02.6]dec-8-ene-3,5-dione (DCD) and ethanolamine (EA). For this purpose, a mixture of ethanolamine, triethanolamine (TEOA), and anhydrous ethanol (EtOH) was added dropwise to the ethanolic solution of 4,10-dioxatricyclo[5.2.1.02,6] dec-8-ene-3,5-dione over 2 h at 0–2 °C. The reaction was carried out at 85–90 °C with constant stirring under argon for 3.5 h. The mixture was cooled down to room temperature, then to −4 °C, and left at this temperature for 12 h. The obtained product was isolated from the reaction mixture by filtration under reduced pressure. The obtained white powder was then washed with isopropanol and vacuum dried. The product, 4-(2-hydroxyethyl)-10-oxa-4-azatricyclo [5.2.1.02.6] dec-8-ene-3,5-dione, was obtained in the form of a white solid. The yield of this step ranged from 70 to 83%.

Step 3: 1-(2-hydroxyethyl)-1H-pyrrole-2,5-dione (HPD) was obtained by a reaction of 4-(2-hydroxyethyl)-10-oxa-4-azatricyclo[5.2.1.02.6]dec-8-ene-3,5-dione (HAD) with hydroquinone as an antioxidant in the xylene medium. The reaction was carried out at the boiling point of xylene (130 °C) with constant stirring under argon for 3 h. Then, the by-product (furan) was distilled off. The remaining solution was filtered under reduced pressure. The filtrate was then cooled to room temperature, then to −4 °C, and left at this temperature for 12 h. The product was isolated from the reaction mixture by filtration under reduced pressure, and then the obtained white powder was washed with xylene and dried in vacuo. The product, 1-(2-hydroxyethyl)-1H-pyrrole-2,5-dione, was a beige powder. The yield of this step ranged from 89 to 98%.

Step 4: (Hydroxymethyl)-10-oxatricyclo[5.2.1.02.6]dec-8-ene-3,5-dione-2-aminoethanol (HODA) was prepared by the reaction of 1-(2-hydroxyethyl)-1H-pyrrole-2,5-dione (HPD) and furfuryl alcohol (FA) in a toluene environment. The reaction was carried out at 80 °C under constant stirring under argon for 24 h. The mixture was then cooled to room temperature, and the product was separated from the reaction mixture by filtration under reduced pressure. The resulting powder was washed with diethyl ether and dried in vacuo. The product obtained was a beige powder. The yield of this step ranged from 84 to 90%.

The scheme of each step, the structure, and FTIR, ^1^H NMR, and ^13^C NMR spectra of the obtained DCD, HAD, HPD, and HODA are shown in the supplementary data (Figures S2–S5, S6–S9, S10–S13, and S14–S17).

The obtained adduct of the Diels–Alder reaction (HODA) was used for the preparation of the self-healing urethane acrylates.

For this purpose, 1-(Hydroxymethyl)-10-oxatricyclo [5.2.1.02.6] dec-8-ene-3,5-dione-2-aminoethanol (HODA) and dichloromethane were put into a round-bottom flask equipped with a dropper, thermometer, and magnetic stirrer. Then, dibutyltin dilaurate (DD) was added as a catalyst. The solution was purged with argon gas. Then, isophorone diisocyanate (IPDI) was added dropwise for 1 h with constant stirring. The reaction was carried out at 25–35 °C under constant stirring in an inert gas atmosphere for 5–24 h. Then, a solution of polyethylene glycol (PEG) and dibutyltin dilaurate in dichloromethane was added. The mixture was heated to 65–85 °C and stirred for 2–5 h. A solution of (meth)acrylate (HR(M)A) and dibutyltin dilaurate in dichloromethane was then added dropwise. Then, the mixture was cooled to 50–60 °C. The reaction was continued until the band at 2270 cm^−1^, characteristic of the -NCO group, disappeared (ATR-FTIR control). The types and amounts of the reagents used are summarized in Table 1.

### 2.3. General Analytical Methods to Confirm the Structure

The structure and purity of the obtained compounds were confirmed by spectroscopic methods (^1^H NMR, ^13^C NMR, and ATR-FTIR).

The ^1^H and ^13^C nuclear magnetic resonance (NMR) spectra were recorded with a BRUKER DPX-400 spectrometer (Billerica, MA, USA) at 400 MHz (^1^H) and 100 MHz (^13^C) in CD_2_Cl_2_ as a solvent. The chemical shifts (δ, ppm) are given relative to TMS used as the internal standard.

The attenuated total reflectance Fourier transform infrared (ATR-FTIR) spectra were measured on a Thermo Scientific Nicolet 380 spectrometer (Waltham, MA, USA) equipped with an ATR diamond plate. The spectra were recorded in transmission mode in the range of 4000–400 cm^−1^ at a resolution of 4 cm^−1^ and analyzed using the Omnic program version 7.3.

### 2.4. Characteristics of the Photopolymerization Process and Properties of Cured Coatings

The UV-curing process of synthesized urethane (meth)acrylates was monitored by Fourier transform infrared spectroscopy (FTIR) and photodifferential scanning calorimetry (photo-DSC).

FTIR spectroscopy was carried out using a Nicolet iS5 instrument (Thermo Fisher Scientific, Waltham, MA, USA). The resolution is 4 cm^−1^, and the scanning range is 400–4000 cm^−1^. The recording interval of the spectrum was 10 s. Series real-time IR (RT-IR) was used to determine the conversion of (meth)acrylate double bonds and determine the photopolymerization rate. This spectroscopic technique permits in situ monitoring of the chemical processes by mimicking the disappearance of the characteristic bonds of the reactive monomer subjected to UV exposure [25]. The mixture of urethane (meth)acrylate resins and an initiator was placed in a mold made from glass slides and spacers measuring 15 mm in diameter and 0.2 mm in thickness. The samples were placed in the compartment of a Fourier transform infrared spectrometer and were simultaneously exposed to a UV light source (a mercury UV lamp, 36 W, 280–400 nm, 10 mW/cm^2^) and an IR analyzing light beam. The absorbance change of the (meth)acrylate double bond (C=C) peak area was correlated to the extent of polymerization. The conversion degree (DC) can be expressed by the following relationships: DC (%) = (A_0_ − A_t_)·100/A_0_, where A_0_ is the initial peak area before irradiation and A_t_ is the peak area at time t. The photopolymerization rate (R_p_) was calculated by the following relations: R_p_ = dDC/d_t_, where t is the time of irradiation [26].

The UV-curing process of synthesized urethane (meth)acrylates was isothermally monitored (at 25 °C) in a nitrogen atmosphere for 10 min by means of a photo-DSC apparatus (Q100, TA Instruments, New Castle, DE, USA) equipped with the Omnicure S2000 UV light emitter (280–480 nm, 200 mW/cm^2^; Excelitas Technologies, Waltham, MA, USA), providing the first hint about the photoreactivity of the obtained systems. A polymerization solution was composed of urethane (meth)acrylate resin and 3 wt.% of the photoinitiator.

### 2.5. Preparation of Coating Compositions, Cured Films, and Characteristics of Cured Films

The coating compositions were formulated using the synthesized urethane (meth)acrylate resin (after evaporation of the solvent in which the synthesis was carried out) and 3 wt.% of the photoinitiator (ethyl(2,4,6-trimethylbenzoyl)-phenyl phosphinate, Omnirad TPOL, IGM Resins). The components were stirred together under dark conditions until a homogeneous mixture was obtained. Subsequently, the curing solution was applied to the glass substrates by means of a gap applicator (120 µm). The polymeric film was cured under a light source (UV lamp, Aktiprint-mini 18-2, type: UN50029, Technigraf GmbH, Grävenwiesbach, Germany) at room temperature and irradiated under UV light with an intensity of 200 mW/cm^2^ to dryness.

The following tests were performed in order to evaluate the properties of the cured coatings: tack-free time, pendulum hardness test, adhesion, gloss, and yellowness index. Tack-free time was measured as a surface cure time according to ISO 9117. This is the time at which the coating is deemed to have properly adhered and has achieved the final technical parameters. The hardness of coatings was tested using Persoz pendulum hardness on the glass substrate (TQC Sheen, Capelle aan den IJssel, The Netherlands) according to ISO 1522 standard. Adhesion to glass substrates was evaluated according to PN-EN ISO 2409 (crosscut method; BYK, Wesel, Germany). Gloss was measured by the spectrometer GLS (SADT Development Technology Co., Ltd., Beijing, China) according to ASTM D523. The yellowness index is a number calculated from spectrophotometric data that describes the change in color of test samples. This parameter was measured according to ASTM E313 using a precision colorimeter, NH-145 (3NH Technology Co., Ltd., Shenzhen, China).

### 2.6. Characteristics of Self-Healing Properties

The thermally reversible mechanism was monitored using FTIR spectroscopy, DSC, and TG instruments, while the self-healing properties were observed using an optical microscope.

**Figure 1:**
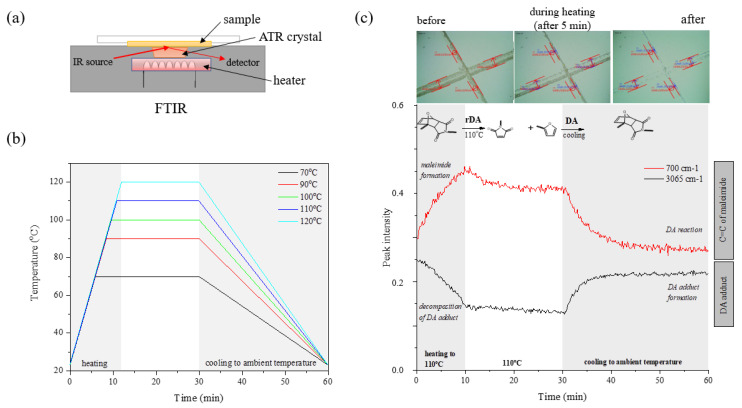
The scheme of the conducted experiment (**a**), the temperature program used in the research (**b**), and the diagram about the interpretation of the results on the relative heights of the peaks in response to temperature changes and chemical reactions (**c**).

FTIR spectroscopy was also conducted to study the thermally reversible mechanism of the DA structures of cured coatings. FTIR examinations were conducted on a Nicolet iS5 instrument (Thermo Fisher, Waltham, MA, USA). The resolution is 4 cm^−1^ and the scanning range is 400–4000 cm^−1^. The recording interval of the spectrum was 10 s. The apparatus was equipped with an ATR attachment and a heating function. The sample (in the form of a hardened coating) was placed on the spectrophotometer attachment, and then the heating was turned on to the set temperature and the FTIR spectra were recorded. The temperature was maintained for 30 min of the cycle, and after that time, the sample was cooled to ambient temperature for the remaining 60 min of the cycle. The samples were tested at 70, 90, 100, 110, and 120 °C with a heating ramp of 8 °C/min. In this way, three-dimensional FTIR spectra were obtained as a function of time and temperature. Figure 1 shows a scheme of the conducted experiment (Figure 1a), the set temperature program (Figure 1b), and information on the interpretation of the test result (Figure 1c). To study the effect of temperature on reversible properties, the peaks of 700 cm^−1^ associated with C=C from maleimide and 3065 cm^−1^ assigned to the presence of the DA adduct were studied in detail.

The DSC test was also used to determine the thermally reversible mechanism of the DA structure of cured coatings. The study was performed on a DSC apparatus (Q100, TA Instruments, New Castle, DE, USA). Aluminum pans were used to seal the sample weights in the range of 7–10 mg. Measurements were carried out in the heating–cooling–heating cycle in the temperature range of −90–300 °C with a heating ramp of 10 °C/min.

Thermal stability for all obtained prepolymers was also investigated. The thermogravimetric (TG) analysis was determined on a Netzsch Proteus Thermal Analysis TG 209 F1 Libra apparatus (Selb, Germany) under an oxidizing atmosphere (nitrogen flow (as protective gas) of –10 cm^3^/min and airflow of –25 cm^3^/min) on the temperature range 25–1000 °C in alumina crucibles (Al_2_O_3_).

Optical microscopy (OM) was used to observe the self-healing properties of the coatings. The research was carried out using an optical microscope (Delta Optical, with MC500-W3 5 MP camera). The camera attached to the microscope was used to capture images at magnifications of 40×. The microscope was equipped with a heating stage (Linkam LTS420, Linkam Scientific Instruments Ltd., Redhill, UK), which heated the sample to the set temperature (70–120 °C, with a heating ramp of 8 °C/min). The test samples were prepared as thin films (120 µm) on a microscope slide. Subsequently, the samples were cured using UV radiation and scratched. The scratches on the cured coatings were created in the form of cut lines by quick manual scratching and with a #11 scalpel blade. The samples prepared in this way were placed on the heating table and heated to the set temperature, kept at the set temperature for 30 min, and then cooled to the ambient temperature. The entire cycle lasted 60 min. The results were recorded in the form of photos taken every 60 s, on which measurements were made in four places. The test was repeated 3 times. The result is the arithmetic mean of all measurements and is expressed as a percentage.

## 3. Results and Discussion

### 3.1. Synthesis and Characterization of UA-DA Resins

**Figure 2:**
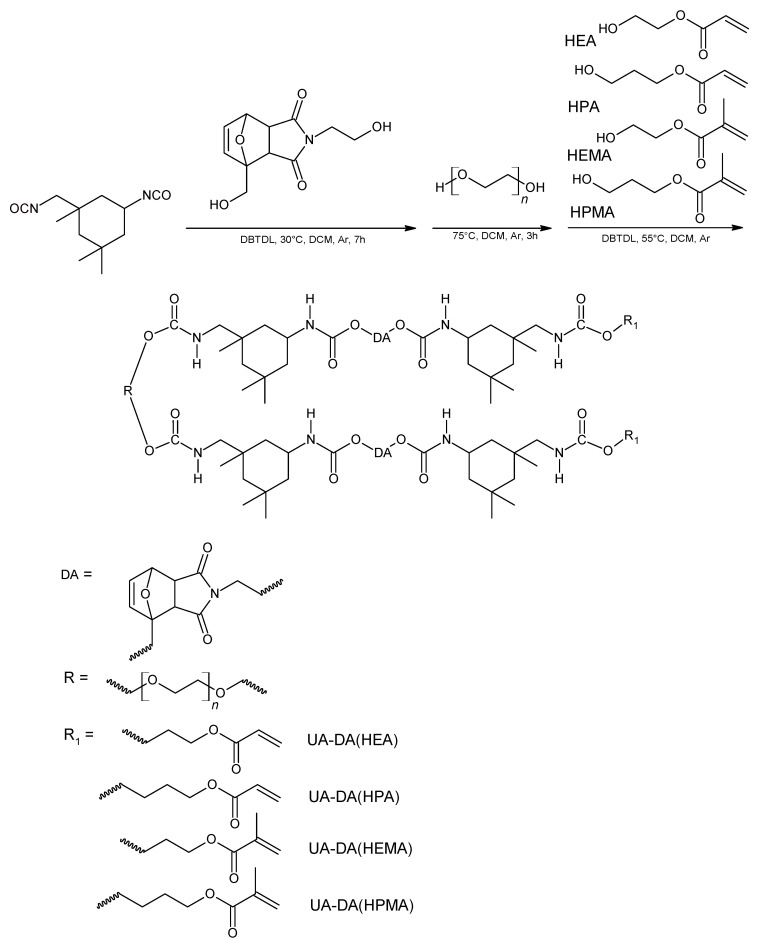
Scheme of the synthesis of the UV-curable urethane (meth)acrylates based on the DA structure (UA-DA) with various (meth)acrylate groups (HEA, HPA, HEMA, and HPMA).

**Figure 3:**
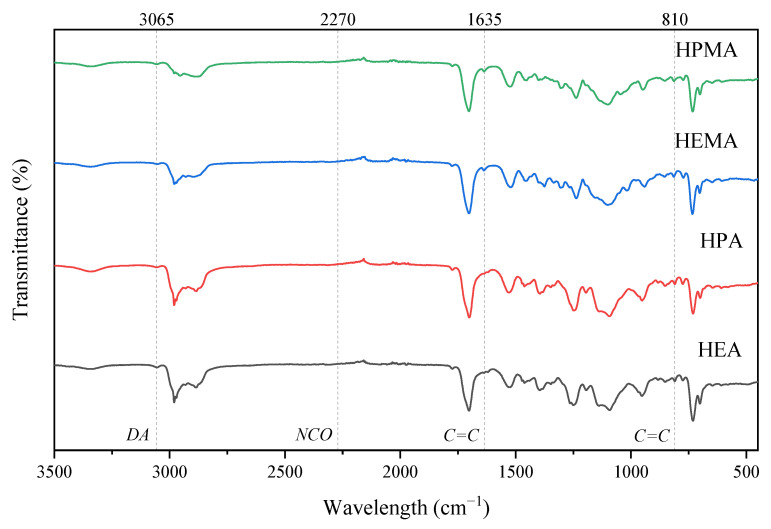
FTIR spectra of urethane (meth)acrylates obtained.

Firstly, urethane (meth)acrylate containing the DA structure (UA-DA) was prepared to take into account the reaction conditions as previously reported [21]. Figure 2 shows the shortened synthesis route of obtained UA-DA resins. In the first step, HODA hydroxyl groups react with IPDI isocyanate groups to form a urethane group. Subsequently, upon the addition of polyethylene glycol, further isocyanate groups react with the PEG hydroxyl groups. In the last step, hydroxyacrylates or hydroxymethacrylates with different chain lengths (C2–C3) were added, which means the resin can be cured by UV irradiation. Thus, upon the addition of the hydroxy(meth)acrylate, the two extreme isocyanate groups react with the hydroxyl groups of the (meth)acrylate to form more urethane bonds. Figure 3 shows the FTIR spectra of all of the UA-DA products after synthesis. The whole synthetic route for the preparation of resins and the NMR and FT-IR spectra taken during the multistep reaction are summarized in the supplementary data (Figures S1–S29). Figure 3 shows the signals confirming the creation of the desired structures. The course of the reaction for the preparation of urethane (meth)acrylates was monitored using the infrared spectroscopy method, observing the characteristic absorption band at 2270 cm^−1^ for the isocyanate group. The most obvious change in the spectrum, confirming that all of the isocyanate groups have reacted, is the disappearance of this band. Moreover, the structure of the compounds obtained was confirmed based on the analysis of other absorption bands, including the typical peaks of polyurethane: the weak band at ca. 3350 cm^−1^ was assigned to v_N–H_, the strong bands at ca. 2850–3000 cm^−1^ were assigned to v_C–H_, the strong bands at ca. 1700 were assigned to v_C=O_, the strong bands at ca. 1520 cm^−1^ were assigned to v_C-N_, and the bands at ca. 1170 were assigned to v_C-O-C_. There are also visible vibrations, characteristic of the Diels–Alder adduct, originating from the C=C bond occurring at 3065 cm^−1^ and characteristic vibrations of the double bond from (meth)acrylate at 1635 and 810 cm^−1^. Therefore, it can be confirmed that the obtained urethane (meth)acrylates containing DA bonds have been synthesized successfully. The differences in the IR bands for the various urethane (meth)acrylates obtained were negligible and resulted from differences in structures.

**Figure 4:**
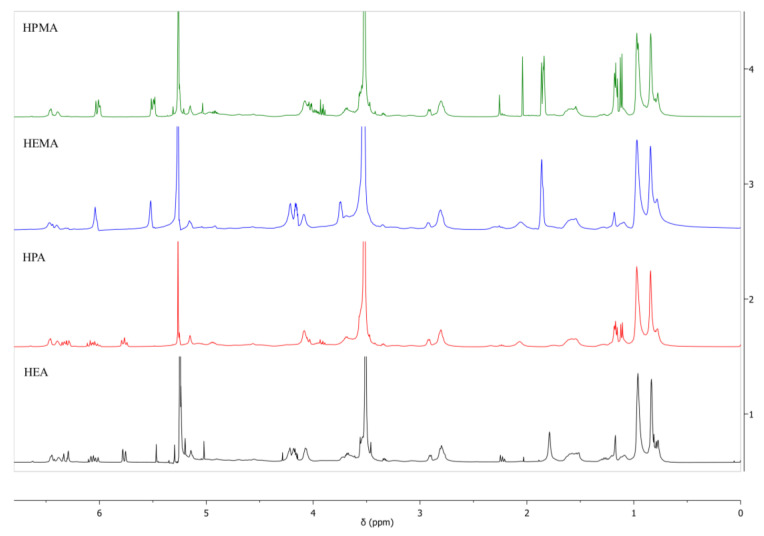
^1^H NMR spectra of urethane (meth)acrylates obtained.

The structure of the obtained urethane (meth)acrylates was also confirmed based on ^1^H NMR spectra. The individual results are presented in the supplementary materials (Figures S22–S25). A comparison of the ^1^H NMR spectra of the obtained urethane (meth)acrylates is shown in Figure 4.

### 3.2. UV-Curing Mechanism of UA-DA Resins

**Figure 5:**
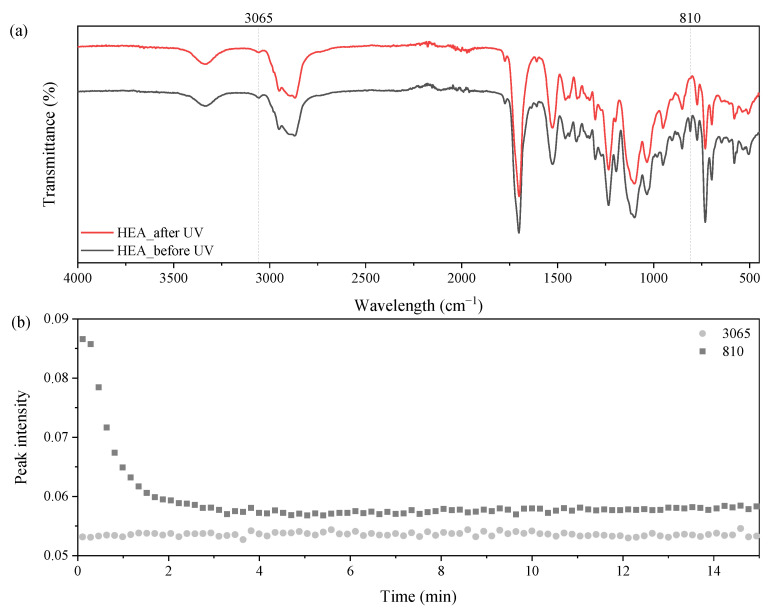
FTIR spectra of UA-DA(HEA) resin (**a**) before and after UV irradiation; (**b**) selected peaks’ intensity during UV irradiation time.

**Figure 6:**
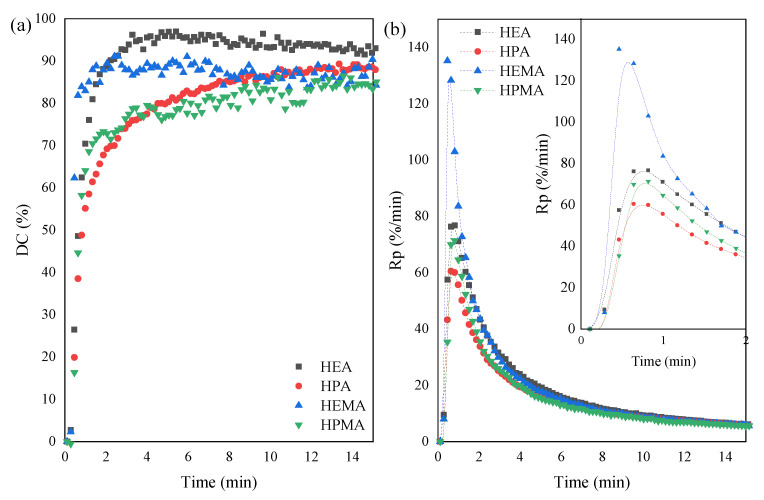
Photopolymerization process of obtained UA-DA resins monitored by FTIR; (**a**)—conversion degree, and (**b**)—photopolymerization rate during UV exposure.

**Figure 7:**
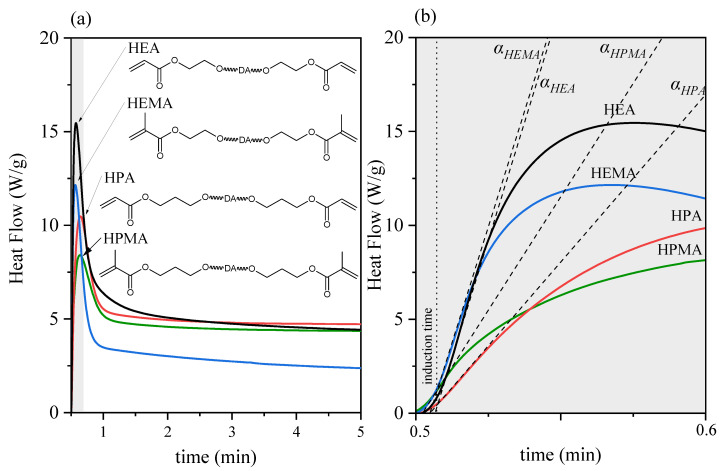
Photopolymerization process of obtained UA-DA resins monitored by photo-DSC; (**a**)—Heat flow during UV exposure; (**b**)—enlargement of a fragment of the Figure 7a.

The photopolymerization process of photoreactive compositions based on the obtained resins and 3% wt. of the radical photoinitiator was followed by FTIR, and the results are shown in Figure 5, Figure 6 and Figure 7. Figure 5 shows the FTIR spectrum of the composition based on UA-DA(HEA) resin before and after UV irradiation. It can be easily seen that the peak intensity at 810 cm^−1^ decreased significantly after UV irradiation. It is also worth emphasizing that the peak intensity of the DA adduct at 3065 cm^−1^ remained unchanged, which indicates that UV curing did not affect the DA structure. The same results were obtained for the other resins, and the spectra before and after UV irradiation are presented in supplementary data (Figure S26).

As shown in Figure 6 and Figure 7 and Table 2, the highest conversion of C=C double bonds was achieved by resins with HEA and HEMA, obtaining 97 and 91%, respectively. However, the resins terminated with longer carbon chains derived from HPA and HPMA and obtained a slightly lower conversion, which was 89 and 86%, respectively. The conversion value slightly increases until a plateau is reached, which is understood as the end of the curing process. The resins with shorter polymer chains (HEA and HEMA) reach a plateau faster than resins with longer chains (HPA and HPMA), whose conversion slowly increases over the entire exposure time. As mentioned in previous literature reports [21], the 100% conversion of acrylate double bond C=C can hardly be reached during UV curing, which can be explained by the vitrification of polymer: in the glassy state, molecular chains are limited in their ability to mobilize and diffuse to meet the radicals, so the UV-curing system is not able to reach the theoretical Tg with 100% conversion. It is also worth noting that the acrylates had a higher degree of conversion than their methacrylate counterparts. The additional methyl group of methacrylates may constitute a spherical hindrance and limit the movement of radicals. The situation is slightly different in the case of analyzing the rate of photopolymerization. On the basis of the photo-DSC tests (Figure 7), the inclination angle of the DSC exotherm curve was determined at the beginning of the process, i.e., after the induction period, which corresponds to the process initiation stage. Short-chain methacrylate (HEMA) photopolymerized the fastest, and the slowest was acrylate with a longer carbon chain (HPA). The results are analogous to those determined by the FTIR method, i.e., the rate of process initiation is higher for acrylates because, at the beginning of the process, the curve runs at a greater inclination to the time axis. In turn, the time to achieve maximum heat flow was longer for methacrylates. The kinetic parameters of the photopolymerization process can be helpful in understanding the self-healing of coatings in more detail.

### 3.3. Properties of Cured Coatings

In order to study the influence of different chain architectures of synthesized urethane (meth)acrylates on the cured coatings, the basic utilitarian properties of cured coatings were determined and are presented in Table 3. All coatings reached surface dryness in a very short time, i.e., up to 6 s. However, we can distinguish the UA-DA(HEA)-based coating, characterized by the shortest tack-free time, which was 3 s. As expected, methacrylate coatings had a higher hardness than acrylate coatings, as well as short-chain polymers (i.e., hydroxyethyl (meth)acrylates) compared to polymers with longer carbon chains (i.e., hydroxypropyl (meth)acrylates). This is possibly related to the higher rigidity of polymer chains containing methacrylate groups or short polymer chains, which we described in our previous works [27,28]. Finally, the obtained urethane (meth)acrylates show excellent properties of cured coatings, such as high hardness, adhesion, perfect gloss, and a low yellowness index.

### 3.4. Thermal Properties of UA-DA Film

Thermal properties were assessed for realistic application and investigation of the thermo-reversibility performance of the cured UA-DA coatings. These properties were evaluated by DSC, TG, and FT-IR.

**Figure 8:**
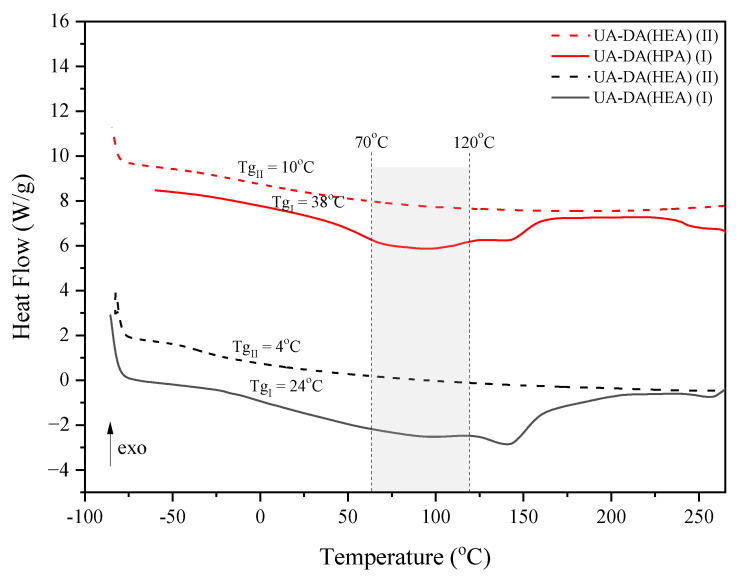
DSC curves during two heating cycles for the UV-cured UA-DA films.

Retro-DA and DA reactions were studied by DSC measurements, applying the heating–cooling–heating cycle for the cured UA-DA(HEA) film and UA-DA(HPA) film (Figure 8; only heating cycles are shown in the figure). The course of the first heating cycle is characteristic of these types of resins with a built-in DA structure [17,21]. In particular, two broad endothermic peaks are visible. The first peak relates to the glass transition of the sample. The data for resin with HEA reveals that the film has a T_g_ at 24 °C in the first heating–cooling cycle. Moreover, as supported by the data reported before [17,21], it is obvious that T_g_ of the film in the second heating–cooling cycle decreased (to around 4 °C) due to the lower crosslink density caused by the incomplete DA reaction during the cooling process. Similar changes were noted for the remaining samples. The second endothermic peak, starting at about 110 °C with a maximum at about 140 °C, concerns the rDA reaction [21]. The decrease in the intensity of this peak in the second heating cycle confirms the above assumptions regarding the change in T_g_. With reference to previous papers on the self-healing of urethane (meth)acrylates with a built-in DA structure, which mention the minimum temperature at which the rDA reaction occurs (60 °C), and an attempt to use the lowest possible temperatures of heating samples in relation to obtaining self-healing properties of cured coatings (i.e., the beginning of endothermic peak related to the rDA reaction, i.e., 110 °C), the following temperature range was used in subsequent studies, i.e., from 70 to 120 °C.

The thermal stability of the obtained urethane (meth)acrylate prepolymers was also compared based on the following properties: onset decomposition temperature and maximum decomposition temperatures (determined from DTG curves), presented in Table 4 and in the figures provided in the supplementary materials (Figures S27–S30). The first stage of decomposition was related to the evaporation of the solvent, methylene chloride. Therefore, it was not included in the stability assessment. For all the urethane (meth)acrylates analyzed, multistep decomposition was observed. The highest onset decomposition temperature was observed for UA-DA(HPA). All the resins obtained were stable at a similar temperature. These compounds start to decompose at temperatures close to 300 °C. No relationship was observed between structure and thermal stability. The next useful parameter for determining the thermal stability of obtained urethane (meth)acrylates is the maximum decomposition temperature for given compounds. For various urethane (meth)acrylates, it was shown that the maximum decomposition temperature falls within the range of 303.9–332.3 °C. The highest temperature was observed for UA-DA(HPA), while the lowest was observed for UA-DA(HEA). These tests confirm good thermal properties in spite of the rDA reaction and thermal stability in the tested range of self-healing of coatings (70–120 °C), i.e., no decomposition of the tested compounds.

**Figure 9:**
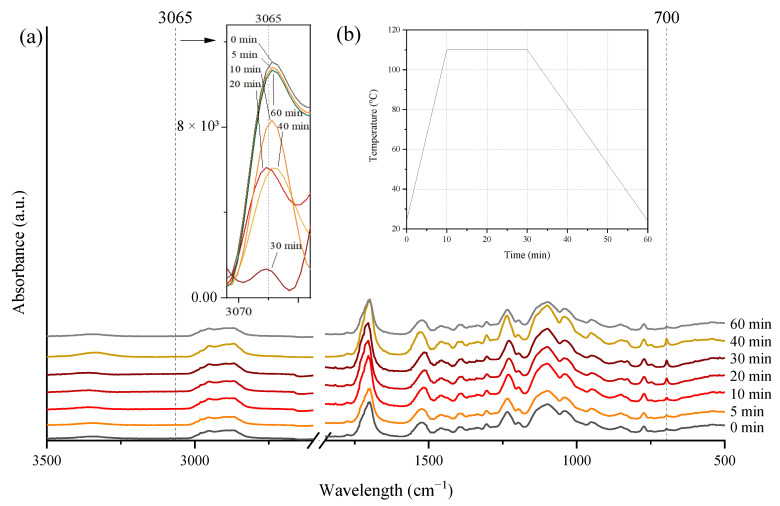
FTIR spectra of the UV-cured UA-DA film during a heating–thermostatic–cooling cycle (on the example of sample UA-DA-HEA at 110 °C)–(**a**); and the temperature program during the test (**b**).

FTIR spectroscopy has also been used to study the thermally reversible mechanism of UA-DA films. For this purpose, the spectra were recorded in real time during the programmed heating–thermostat–cooling cycle using the ART-FTIR heating adapter. Heating temperatures were in the range of 70–120 °C. In the first stage, the possibility of retro-Diels–Alder (rDA) reactions in the cured coating was confirmed on the basis of characteristic peaks previously described in the literature [29]. Thus, Figure 9 shows the evolution of FTIR spectra during a heating–thermostatic–cooling cycle. After heating the sample (for about 10 min), a new peak is observed near 700 cm^−1^, which is attributed to C=C of maleimide [22]. This peak disappears when the sample is cooled to ambient temperature. Over the same time, the peak at 3065 cm^−1^ attributed to the DA adduct shows a complementary trend that is exactly the opposite.

**Figure 10:**
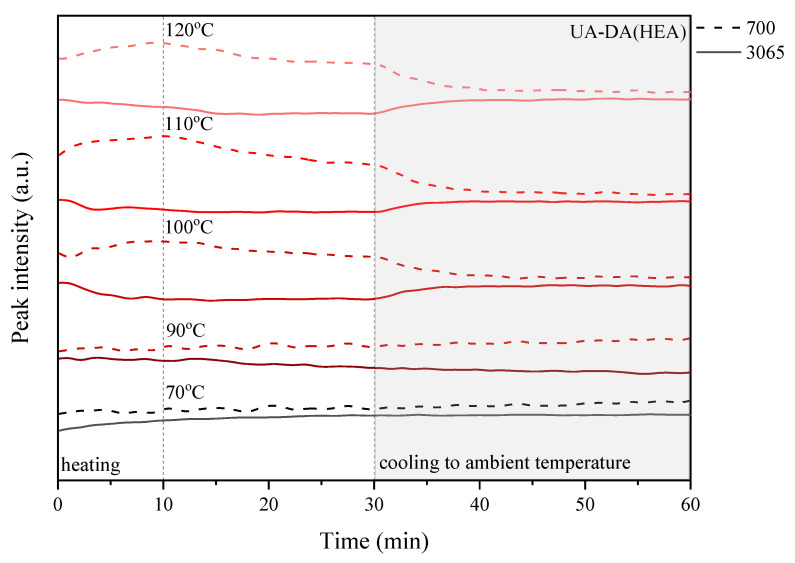
Change in the relative height of selected peaks based on the three-dimensional FTIR spectrum observed during the heating–thermostat–cooling cycle for the UV-cured UA-DA(HEA) sample.

**Figure 11:**
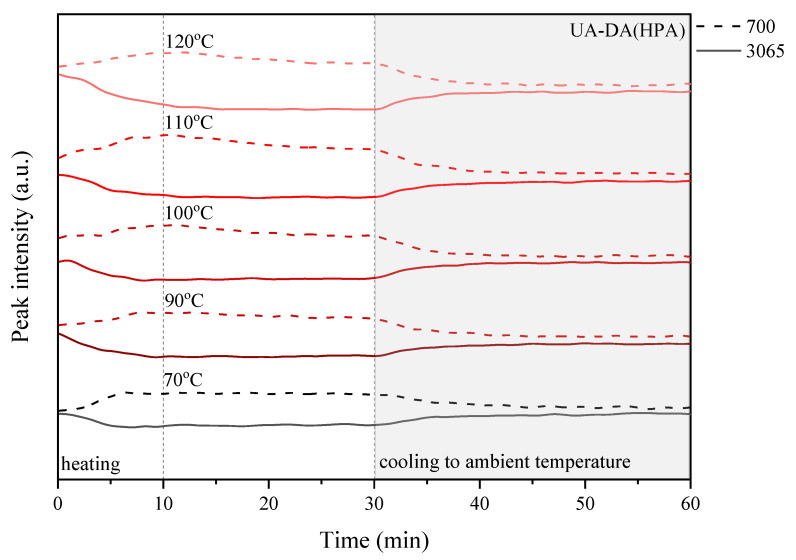
Change in the relative height of selected peaks based on the three-dimensional FTIR spectrum observed during the heating–thermostat–cooling cycle for the UV-cured UA-DA(HPA) sample.

**Figure 12:**
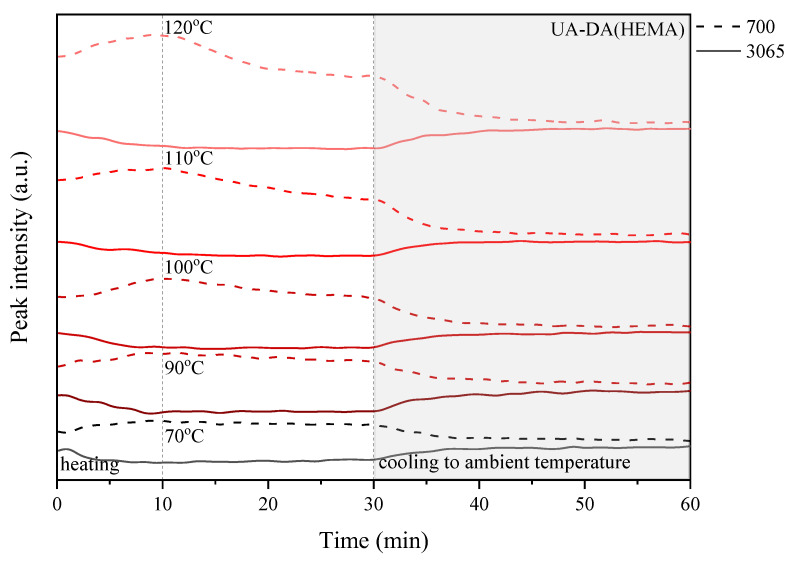
Change in the relative height of selected peaks based on the three-dimensional FTIR spectrum observed during the heating–thermostat–cooling cycle for the UV-cured UA-DA(HEMA) sample.

**Figure 13:**
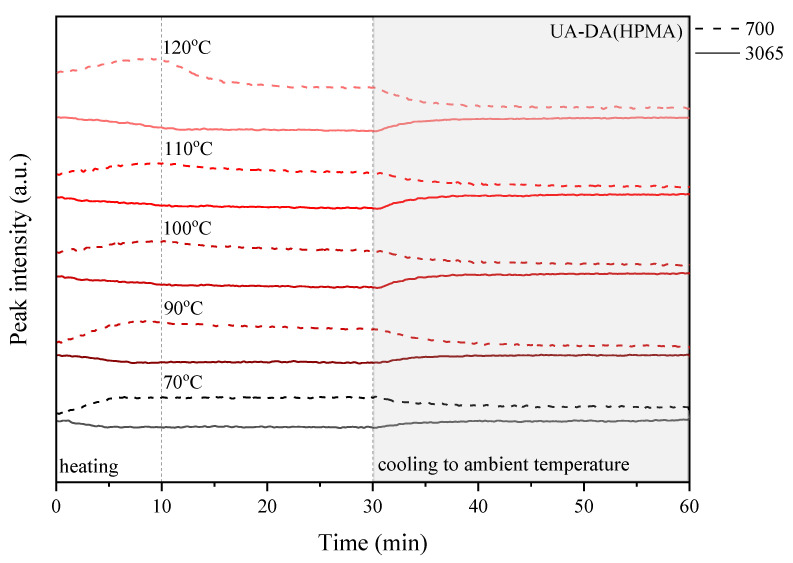
Change in the relative height of selected peaks based on the three-dimensional FTIR spectrum observed during the heating–thermostat–cooling cycle for the UV-cured UA-DA(HPMA) sample.

The above experiment was carried out in various temperature conditions, i.e., in the range of 70–120 °C, in order to study the influence of the chemical structure, in particular the presence of photoreactive groups and the curing process, on the self-healing ability of the coatings. For this purpose, detailed research was carried out considering the peaks responsible for the retro-Diels–Alder (rDA) reaction, i.e., a new peak near 700 cm^−1^, which is related to the absorption of C=C of maleimide, and the peak at 3065 cm^−1^ attributed to the DA adduct. Figure 10, Figure 11, Figure 12 and Figure 13 show the change in the relative heights of the 700 cm^−1^ and 3065 cm^−1^ peaks during the programmed processes: (i) heating to the set temperature, which takes about 10 min; (ii) thermostatic at the set temperature for up to 30 min, i.e., 70, 90, 100, 110, and 120 °C; and (iii) cooling to ambient temperature for 30 to 60 min of the cycle time.

In the studies shown, there was a general trend toward (i) an increase in the 700 cm^−1^ peak, which is caused by an increase in the amount of C=C of maleimide, and a decrease in the intensity of the 3065 cm^−1^ peak, which is caused by the presence of the DA adduct, during heating of the sample and a complementary trend that is the exact opposite; (ii) both peaks stayed the same or their intensities decreased during the thermosetting of the sample.

These changes indicate that a successful retro-Diels–Alder (rDA) reaction occurs in the highly crosslinked coatings. Furthermore, the changes can be monitored by observing the relative height of the selected peaks, which is closely related to the change in temperature (Figure 10, Figure 11, Figure 12 and Figure 13). In the case of the UA-DA(HEA) sample, i.e., a urethane acrylate terminated with a relatively short carbon chain (C3), the expected changes in the level of selected peaks are observed only at a temperature of 100 °C with a slight disappearance of the 700 cm^−1^ peak during thermostatic heating of the sample. This may indicate the occurrence of structural changes unrelated to the DA reaction and self-repair of the coatings. In turn, the UA-DA(HPA) coating, i.e., urethane acrylate with a longer carbon chain (C4), exhibits the expected changes in the relative height of selected peaks at 70 °C, with increasing intensity with increasing cycle temperature and no significant disappearance of the 700 cm^−1^ peak during thermostats. In the case of UV-cured coatings obtained on the basis of appropriate methacrylates, similar changes are observed as in the case of acrylates, with the exception that the HEMA sample maintains the correct trend of increase and disappearance of the tested peaks at 70 °C. The disappearance of the 700 cm^−1^ peak is observed in both samples, at 100 °C for HEMA and at 120 °C for HPA. This discussion is continued below in the context of conducting microscopic studies.

### 3.5. Self-Healing Ability of UA-DA Coatings

The capability of UV-cured coatings to self-heal is generally evaluated by observing the change in the appearance of scratches on their surface. Therefore, a number of experiments were performed at different temperatures to test the self-healing properties of UA-DA films with and without a photoreactive group at the end of the oligomer chains. The healing of thin coatings was investigated by observing the evolution of a surface scratch using an optical microscope (OM) equipped with a heating stage and appropriate software that enabled the experiments to be programmed over time. The samples were placed on a heating stage at the desired temperature, and the scratch width was monitored using the OM. The samples were heated in the temperature range from 70 to 120 °C, and the set temperature was maintained for 30 min, after which the sample was cooled to ambient temperature.

**Figure 14:**
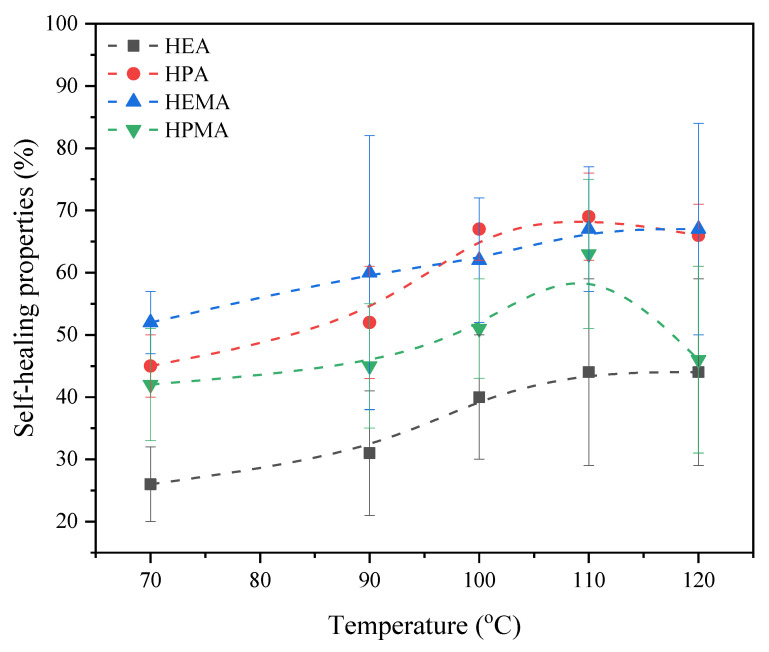
Self-healing properties based on microscopic images of UA-DA films heated to different temperatures.

The results showing the self-healing of the coatings were developed on the basis of microscopic images, on which the width of the cracks was determined before the heating process and in each minute of the cycle lasting a total of 60 min. The tests were developed as a percentage progress of crack healing, which is presented in Figure 14, while microscopic images before and after the process are presented in Table 5. The degree of self-healing of the UV-cured coatings increased with increasing temperature, as expected, but only up to 110 °C. All of the tested coatings achieved the lowest degree of self-healing properties at 70 °C (range: 26–52%) and the highest degree at 110 °C (range: 43–69%). Interestingly, both acrylates and methacrylates with shorter polymer chains (HPA and HEMA) achieved a lower degree of self-healing properties at 120 °C than at 110 °C. The samples tested at 120 °C are characterized by a nonstandard course of the relative peak height of 700 cm^−1^ (based on the three-dimensional FTIR spectrum analysis), which apparently decreases during thermostatic. These changes may indicate a decrease in the number of maleimide C=C bonds. However, taking into account the results of the microscopic examination, it would not be in favor of the retro-Diels–Alder reaction. Despite the confirmation of the thermal stability of the tested samples in thermogravimetric tests, up to about 300 °C, the disappearance of this bond is clearly related to the increased temperature. Nevertheless, at the moment, this mechanism is unknown. A coating based on a short polymer chain urethane acrylate, i.e., UA-DA-HEA, has the lowest degree of self-healing properties regardless of temperature. Among all tested samples, this coating had the highest degree of conversion of unsaturated bonds (97%), achieved in a very short time, and the highest total enthalpy of the photocuring process. In addition, the HEA sample also had a higher hardness compared to its longer-chain acrylate counterpart, HPA (86 for HEA and 58 for HPA), which also translates into the results on the self-healing properties. Furthermore, the DSC thermographs show that the UA-DA (HEA) sample has a clearly smaller endothermic peak in the 70–120 °C range, which was assigned to the rDA reaction rather than the UA-DA (HPA). Therefore, it is concluded that both the results of the photocuring process, i.e., a high degree of conversion of unsaturated bonds and the hardness of the coating, as well as the results of tests carried out at elevated temperatures (FTIR and DSC) indicate the existence of limitations related to the possibility of rDA reactions. The extremely stiff microenvironment and the difficult mobility of carbon chains in order to undergo thermally reversible reactions resulting in coating self-healing are most likely to blame. The remaining tested urethane (meth)acrylates were characterized by a similar degree of self-healing, which was within the measurement error limits of the tested samples. It is also worth mentioning here that the results of self-healing may be related not only to the chemistry of the reaction at elevated temperature but also to the physical parameters of the scratch, i.e., the size of the scratch or the quality of the crack. On the basis of the above studies conducted in terms of micro- and macroscopic characteristics, it is indicated that the chemical structure of photoreactive groups in urethane (meth)acrylates with the DA adduct has an influence on the curing mechanism, resulting in significant self-healing efficiency and self-healing temperature.

## 4. Conclusions

UV-curable self-healing urethane (meth)acrylate (UA) coatings were successfully prepared by a photoinitiated radical process using different (meth)acrylate groups present at the ends of the oligomer chains, which are characterized by excellent properties of cured coatings. In the present work, the influence of the chemical structure of the obtained UAs with a built-in DA adduct on the photocuring process, efficiency, and self-healing temperature was demonstrated. The chemical structure of UA was confirmed by FTIR and NMR spectroscopy. The research on the photocuring process was presented using real-time FTIR and photo-DSC. In turn, self-repair studies were presented, taking into account the macro- and microscopic-level approaches, i.e., research on the thermally reversible mechanism using DSC, TG, and FTIR equipped with a heating attachment as well as an optical microscope equipped with a heating table. The presented research can be the basis for the design of self-healing photoreactive polymers for a wide range of applications. In particular, they can be useful for obtaining cured coatings in a very short time and materials that are impossible to repair or maintain.

## Figures and Tables

**Table 1:** Summary of the amounts of substrates used in the synthesis.

Sample Code	m_HODA_ [g]	m_DD_ [g]	m_IPDI_ [g]	M_PEG_	m_PEG_ [g]	m_DD_ [g]	HR(M)A	m_HR(M)A_	m_DD_ [g]
UA-DA(HEA)	9.40	0.43	17.54	1000	19.73	0.28	HEA	4.58	0.29
UA-DA(HPA)	7.93	0.25	14.75	1000	20.02	0.14	HPA	5.20	0.17
UA-DA(HEMA)	6.51	0.11	12.19	1000	13.70	0.13	HEMA	3.58	0.13
UA-DA(HPMA)	9.07	0.21	16.93	1000	19.03	0.22	HPMA	5.49	0.27

m_HODA_—mass of used 1-(Hydroxymethyl)-10-oxatricyclo [5.2.1.02.6] dec-8-ene-3,5-dione-2-aminoethanol (HODA), m_DD_—mass of used dibutyltin dilaurate (DD), M_IPDI_—mass of used isophorone diisocyanate, M_PEG_—average molecular mass of used PEG, m_PEG_—mass of used polyethylene glycol, HR(M)A—the type of (meth)acrylate used in the synthesis, m_HR(M)A_—mass of used (meth)acrylate.

**Table 2:** Characteristics of the photocuring process of UA-DA resins.

Sample Code	ΔH_total_ (J/g)	t_max_ (s)	α	DC_max_ (%)	Rp_max_ (%/min)
UA-DA(HEA)	466	4.2	67	97	78
UA-DA(HPA)	139	8.4	42	89	61
UA-DA(HEMA)	278	4.2	68	91	135
UA-DA(HPMA)	204	8.4	50	86	71

ΔH_total_—total enthalpy of the photocuring process; t_max_—time to achieve maximum heat flow (the time when there was no radiation was subtracted); α—the angle of inclination of the line, calculated from the slope of the line as tangent a; DC_max_—maximum conversion degree; Rp_max_—the maximum rate of conversions.

**Table 3:** Properties of the cured coatings.

Sample Code	Tack-Free Time (s)	Hardness	Adhesion	Gloss (GU)	Yellowness Index
UA-DA(HEA)	3	86	2.0	215	4.5
UA-DA(HPA)	6	58	1.0	166	5.5
UA-DA(HEMA)	6	190	1.5	140	7.5
UA-DA(HPMA)	6	111	1.5	151	3.6

**Table 4:** Thermal stability of the obtained urethane (meth)acrylates.

Sample Code	T_IDT_ [°C]	T_MDT_ [°C]
UA-DA(HEA)	274.7	303.9
UA-DA(HPA)	307.5	332.3
UA-DA(HEMA)	294.1	326.2
UA-DA(HPMA)	304.7	326.9

T_IDT_—onset decomposition temperature, T_MDT_—maximum decomposition temperature.

**Table 5:** Optical microscopy images of self-healing properties of coatings with scratches on the UA-DA films heated to different temperatures.

Sample Code		70 °C	90 °C	100 °C	110 °C	120 °C
**UA-DA (HEA)**	**before**	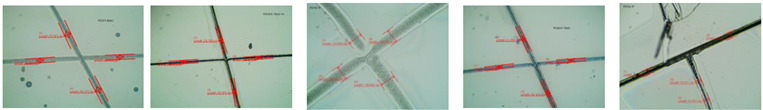 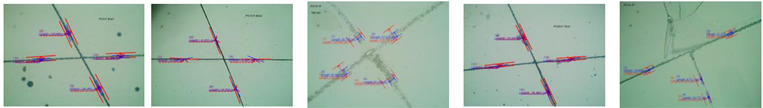				
	**after**					
**UA-DA (HPA)**	**before**	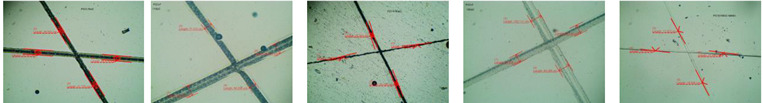 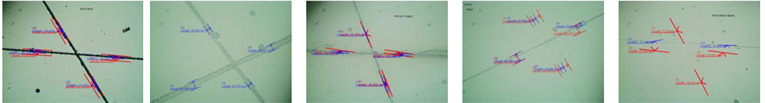				
	**after**					
**UA-DA (HEMA)**	**before**	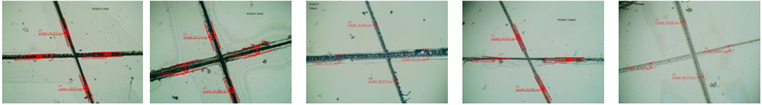 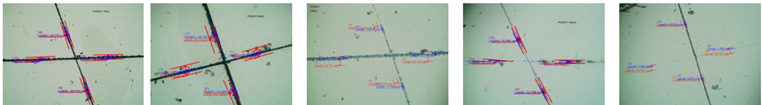				
	**after**					
**UA-DA (HPA)**	**before**	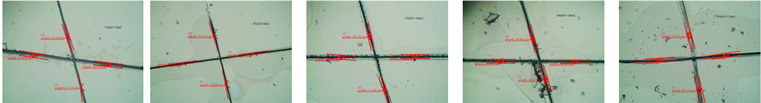 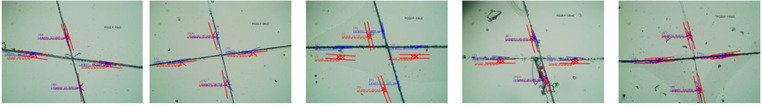				
	**after**					

## Data Availability

Not applicable.

## Supplementary Materials

The following supporting information can be downloaded at: https://www.mdpi.com/article/10.3390/polym15040924/s1, Figure S1: The whole synthetic route of the self-healing urethane (meth)acrylates synthesis; Figure S2: Scheme of the reaction of obtaining DCD; Figure S3: ^1^H NMR DCD spectrum; Figure S4: ^13^C NMR DCD spectrum; Figure S5: ATR-FTIR DCD spectrum; Figure S6: Scheme of the reaction of obtaining HAD; Figure S7: ^1^H NMR HAD spectrum; Figure S8: ^13^C NMR HAD spectrum; Figure S9: ATR-FTIR HAD spectrum; Figure S10: Scheme of the reaction of obtaining HPD; Figure S11: ^1^H NMR HPD spectrum; Figure S12: ^13^C NMR HPD spectrum; Figure S13: ATR-FTIR HPD spectrum; Figure S14: Scheme of the reaction of obtaining HODA; Figure S15: ^1^H NMR HODA spectrum; Figure S16: ^13^C NMR HODA spectrum; Figure S17: ATR-FTIR HODA spectrum; Figure S18: Comparison of FTIR spectra investigating the reaction of receiving UA-DA(HEA) after 0 (blue), 1 (red), and 3 h (green) of reaction time. Comparison of FTIR peaks: NCO stretching at 2270 cm^−1^; Figure S19: Comparison of FTIR spectra investigating the reaction of receiving UA-DA(HPA) after 0 (blue) and 1 h (red) of reaction time. Comparison of FTIR peaks: NCO stretching at 2270 cm^−1^; Figure S20: Comparison of FTIR spectra investigating the reaction of receiving UA-DA(HEMA) after 0 (blue) and 1 h (red) of reaction time. Comparison of FTIR peaks: NCO stretching at 2270 cm^−1^; Figure S21: Comparison of FTIR spectra investigating the reaction of receiving UA-DA(HPMA) after 0 (blue), 1 (red), 4 (green), and 24 h (pink) of reaction time. Comparison of FTIR peaks: NCO stretching at 2270 cm^−1^; Figure S22: ^1^H NMR spectra of UA-DA(HEA). Figure S23: ^1^H NMR spectra of UA-DA(HPA); Figure S24: ^1^H NMR spectra of UA-DA(HEMA); Figure S25: ^1^H NMR spectra of UA-DA(HPMA); Figure S26: FTIR spectra of photoreactive compositions based on obtained resins, before and after UV irradiation; Figure S27: TG and DTG curves of UA-DA(HEA); Figure S28: TG and DTG curves of UA-DA(HPA); Figure S29: TG and DTG curves of UA-DA(HEMA); Figure S30: TG and DTG curves of UA-DA(HPMA).
