# Supplementary material for: Impact of the Chemical Structure of Photoreactive Urethane (Meth)Acrylates with Various (Meth)Acrylate Groups and Built-In Diels–Alder Reaction Adducts on the UV-Curing Process and Self-Healing Properties
Source: Polymers (Basel). 2023 Feb 12;15(4):924. doi: 10.3390/polym15040924 (PMC9967232; doi:10.3390/polym15040924)
Supplement: Supplementary file 1 [file polymers-15-00924-s001.zip › polymers-2127249-supplementary.pdf]

Supporting information

Impact of the Chemical Structure of Photoreactive Urethane (Meth)Acrylates with Various (Meth)Acrylate Groups and Built-in Diels–Alder Reaction Adducts on the UV-Curing Process and Self-Healing Properties

Paulina Bednarczyk *, Karolina Mozelewska, Joanna Klebeko, Joanna Rokicka and Paula Ossowicz-Rupniewska

Department of Chemical Organic Technology and Polymeric Materials, Faculty of Chemical Technology and Engineering, West Pomeranian University of Technology in Szczecin, Piastów Ave. 42, 71-065 Szczecin,
Poland; karolina_mozelewska@zut.edu.pl (K.M.); joanna.klebeko@zut.edu.pl (J.K.); joanna.rokicka@zut.edu.pl (J.R.); paula.ossowicz@zut.edu.pl (P.O.-R.)
* Correspondence: bednarczyk.pb@gmail.com or paulina.bednarczyk@zut.edu.pl

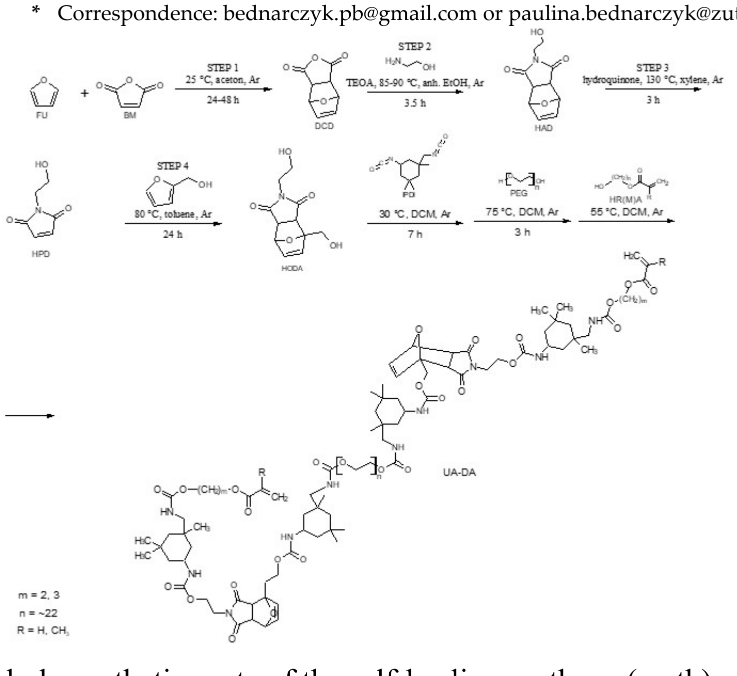

Figure S1. The whole synthetic route of the self-healing urethane (meth)acrylates synthesis

^{1}H, ^{13}C NMR and FT-IR spectra of intermediate products and self-healing polyurethanes

Step 1 – Synthesis of DCD (4,10-dioxatricyclo [5.2.1.02,6] dec-8-ene-3,5-dione)

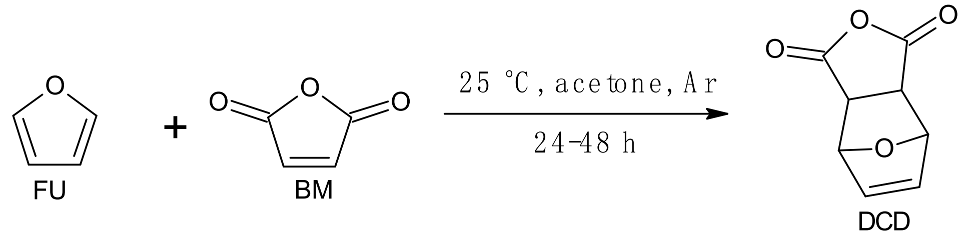

Figure S2. Scheme of the reaction of obtaining DCD

DCD – 4,10-dioxatricyclo [5.2.1.02,6] dec-8-ene-3,5-dione

^{1}H NMR (400 MHz, d$_6$-DMSO, 298 K) δ (ppm) 6.58 (s, 2H), 5.47 (s, 2H), 3.18 (s, 2H); **^{13}C NMR** (400 MHz, d$_6$-DMSO, 298 K) δ (ppm) 48.72, 82.23, 137.01, 169.92; **FT-IR (cm^{-1})** 429, 575, 621, 634, 675, 691, 733, 800, 822, 848, 878, 903, 921, 947, 1002, 1019, 1084, 1145, 1193, 1211, 1230, 1243, 1282, 1310, 1569, 1597, 1779, 1857, 2991, 3002, 3034, 3100.

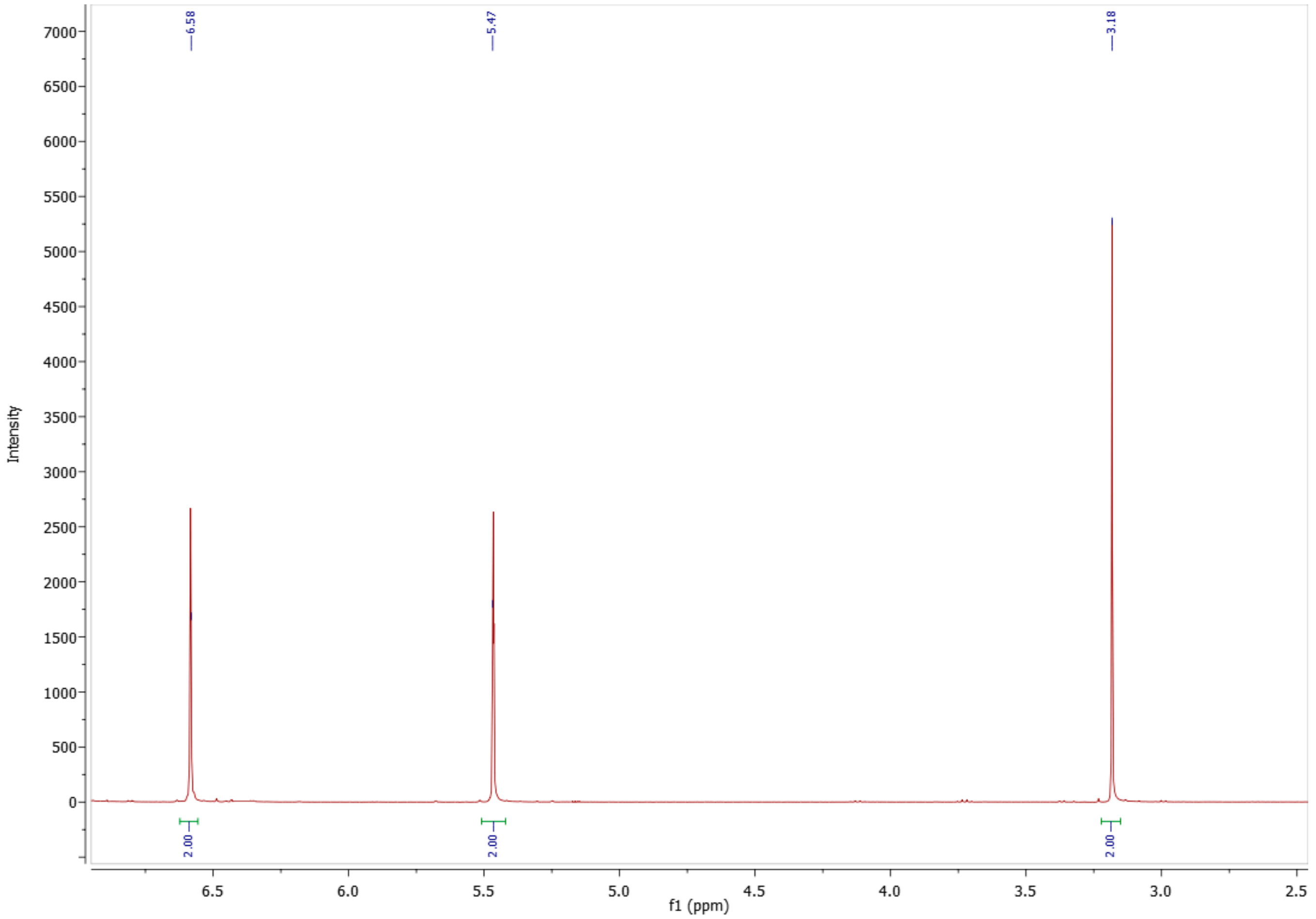

Figure S3. ^{1}H NMR DCD spectrum

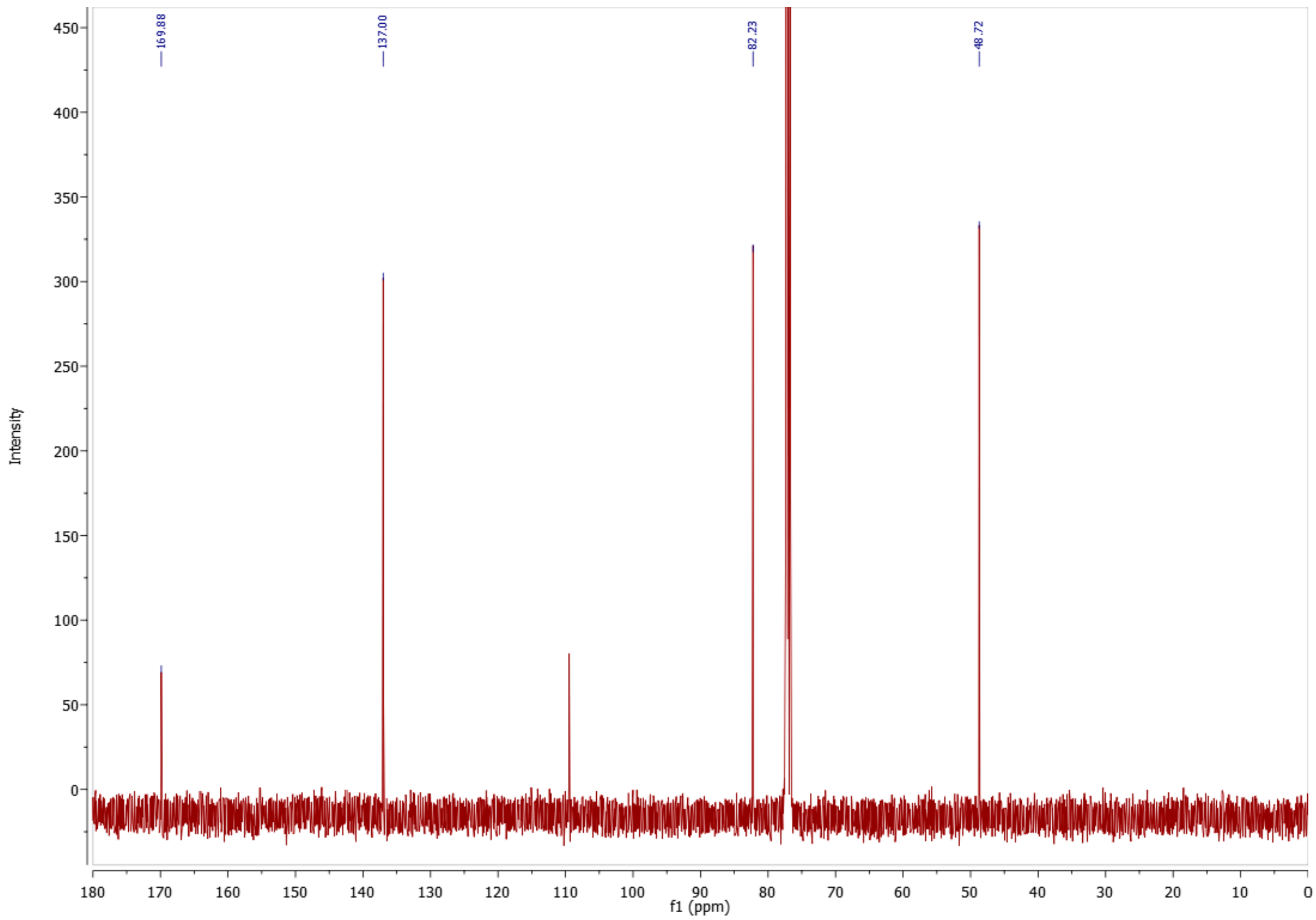

Figure S4. ^{13}C NMR DCD spectrum

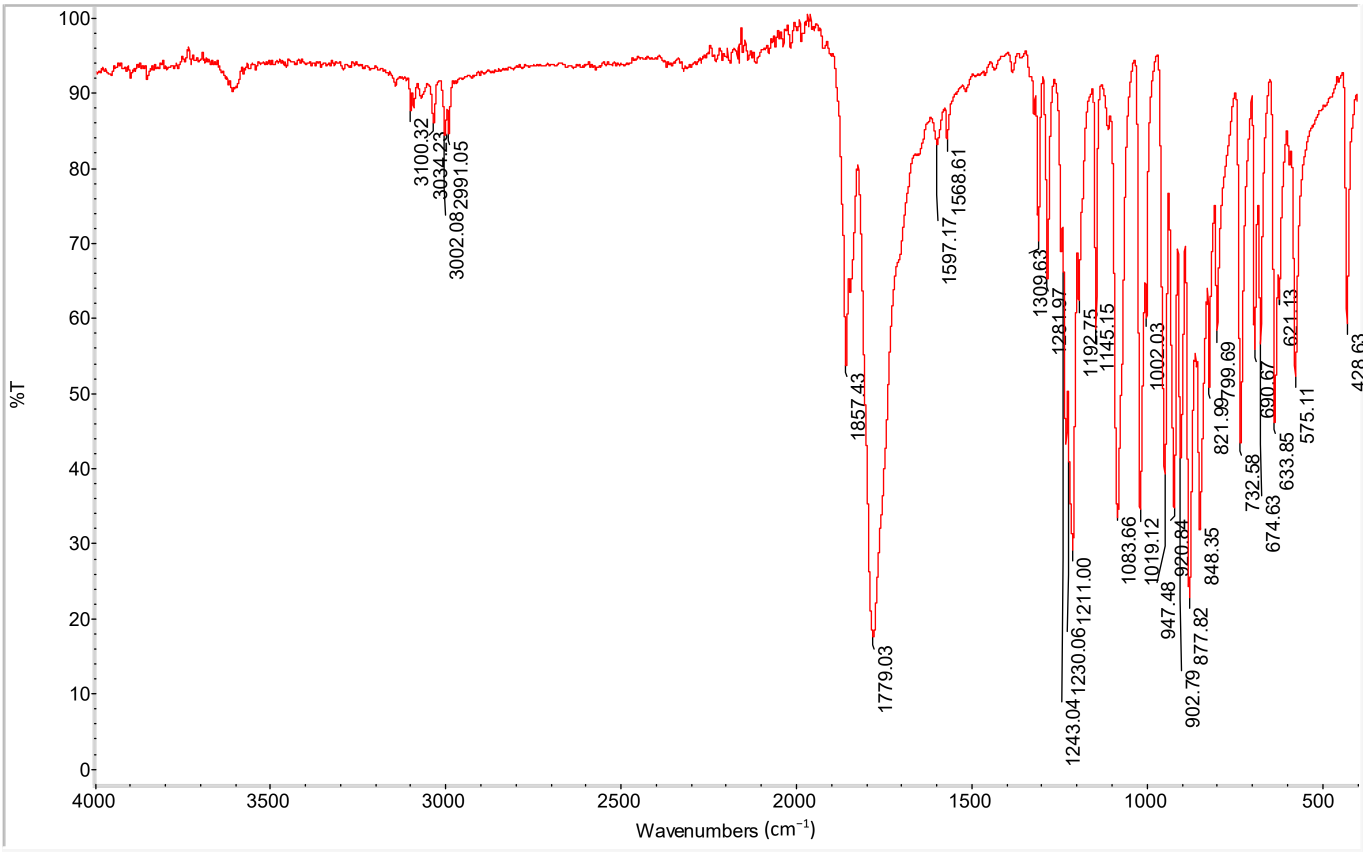

Figure S5. ATR-FTIR DCD spectrum

Step 2 – Synthesis of HAD (4-(2-hydroxyethyl)-10-oxa-4-azatricyclo[5.2.1.02.6]dec-8-ene-3,5-dione)

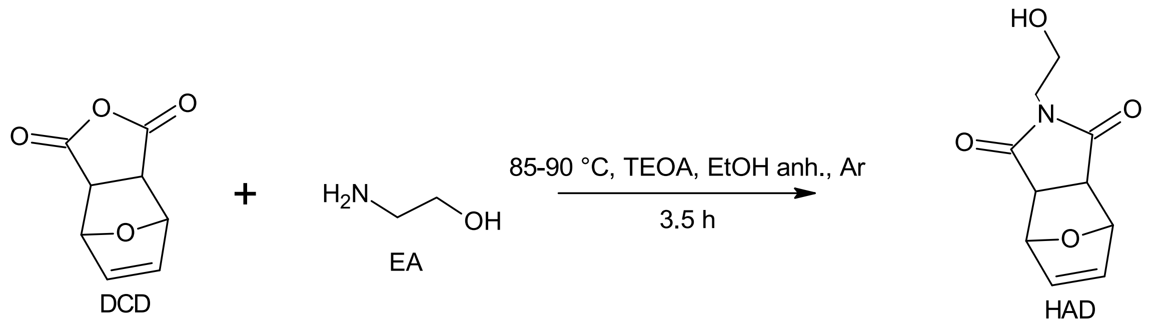

Figure S6. Scheme of the reaction of obtaining HAD

HAD (4-(2-hydroxyethyl)-10-oxa-4-azatricyclo[5.2.1.02.6]dec-8-ene-3,5-dione)

^{1}H NMR (400 MHz, $CDCl_3$, 298 K) δ (ppm) 6.53 (s, 2H), 5.28 (s, 2H), 3.77 (t, 2H) , 3.67 (t, 2H), 2.90 (s, 2H); **^{13}C NMR** (400 MHz, $CDCl_3$, 298 K) δ (ppm): 176.83; 136.53; 80.97; 60.09; 47.50; 41.71; **FT-IR (cm^{-1})** 430, 487, 565, 596, 628, 654, 705, 722, 772, 807, 850, 874, 917, 938, 959, 1014, 1033, 1053, 1100, 1156, 1168, 1220, 1269, 1317, 1335, 1387, 1405, 1435, 1470, 1683, 1766, 2895, 2932, 2973, 2993, 3008, 3098, 3473.

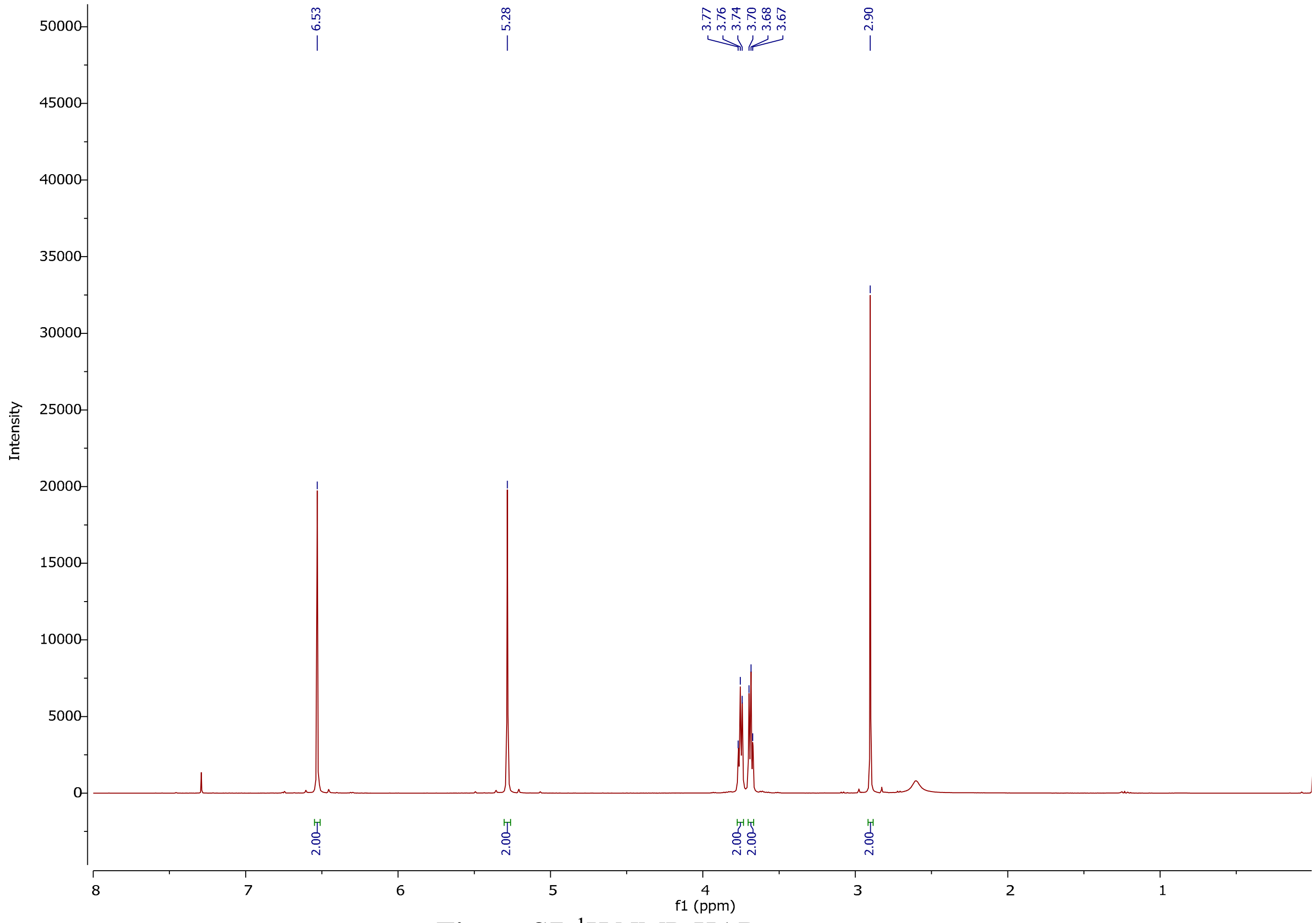

Figure S7. ^{1}H NMR HAD spectrum

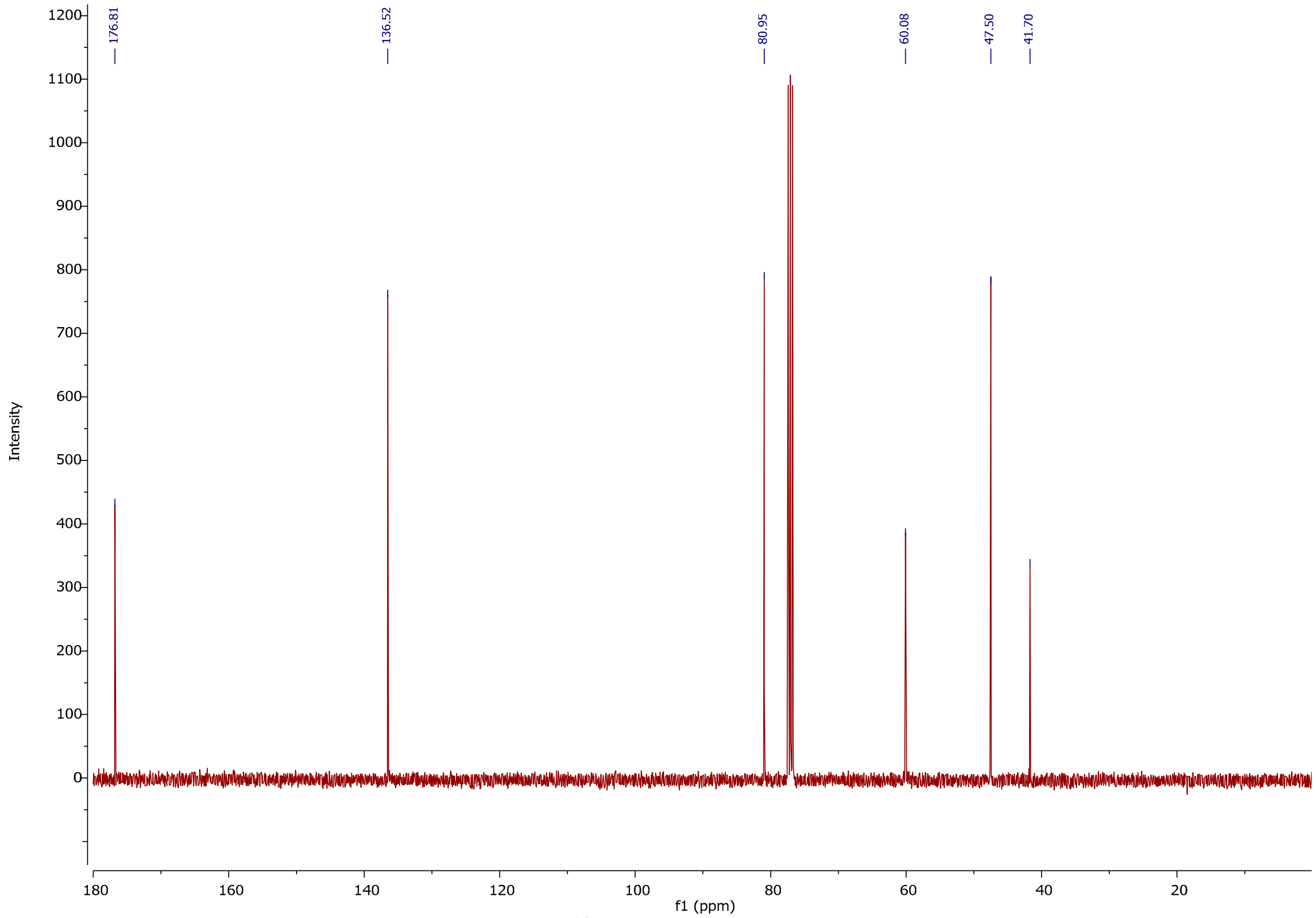

Figure S8. ^{13}C NMR HAD spectrum

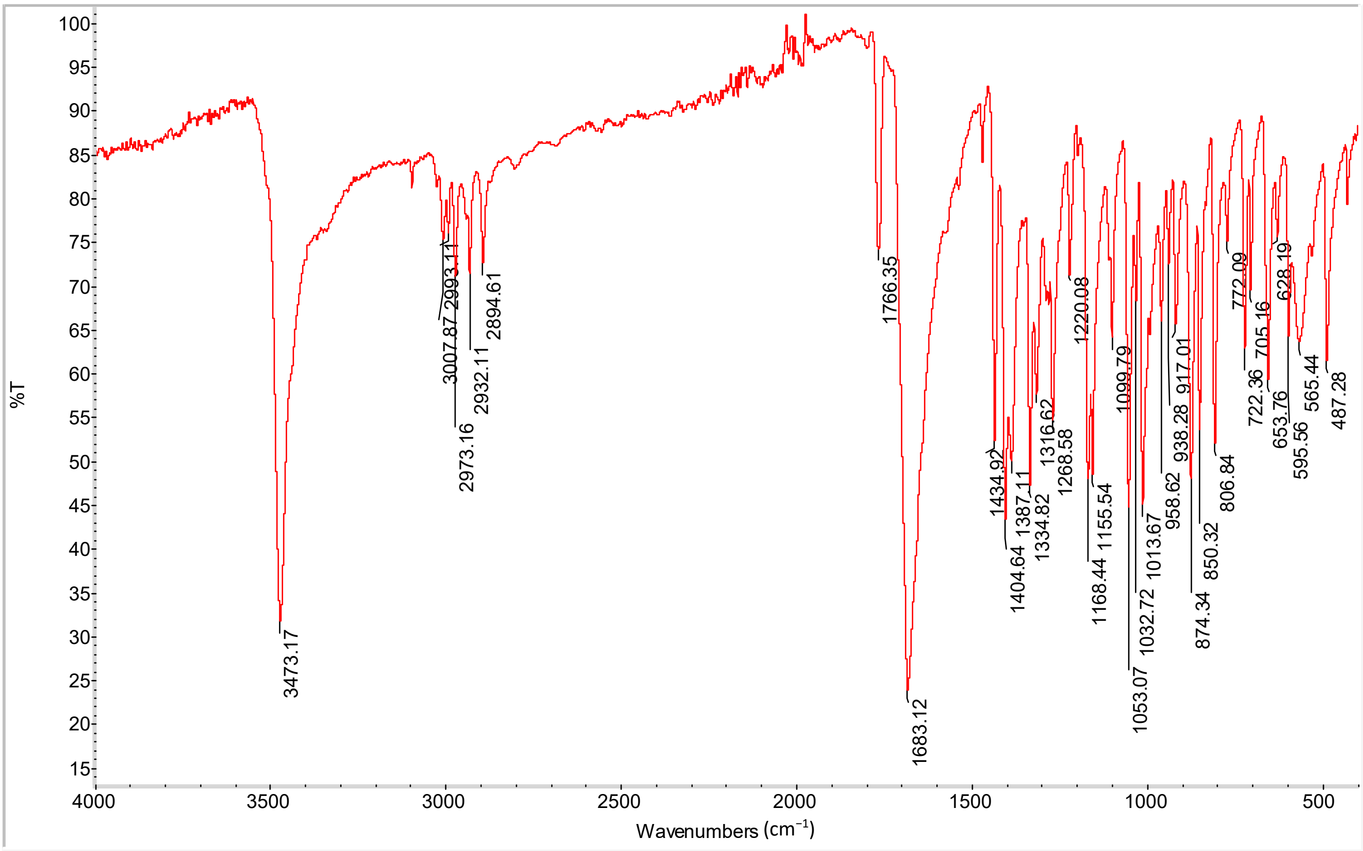

Figure S9. ATR-FTIR HAD spectrum

Step 3 – Synthesis of HPD (1-(2-hydroxyethyl)-1H-pyrrole-2,5-dione)

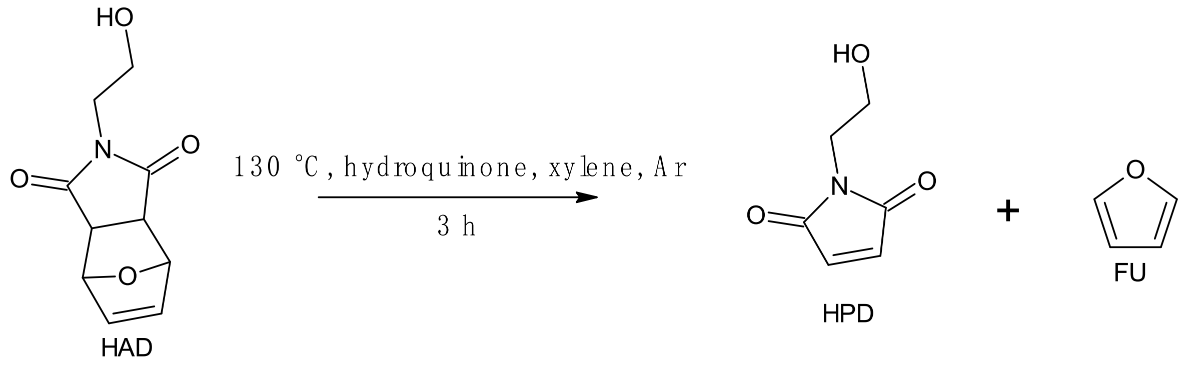

Figure S10. Scheme of the reaction of obtaining HPD

HPD (1-(2-hydroxyethyl)-1H-pyrrole-2,5-dione)

^{1}**H NMR** (400 MHz, $CDCl_3$, 298 K) δ (ppm) 6.75 (s, 2H), 3.77 (t, 2H), 3.71 (q, 2H); ^{13}**C NMR** (400 MHz, $CDCl_3$, 298 K) δ (ppm): 171.21; 134.24; 60.48; 40.52; **FT-IR (cm^{-1})** 505, 573, 608, 692, 825, 852, 930, 972, 1052, 1066, 1093, 1157, 1259, 1321, 1362, 1387, 1403, 1441, 1585, 1707, 1750, 1764, 2880, 2932, 2960, 3114, 3257, 3446.

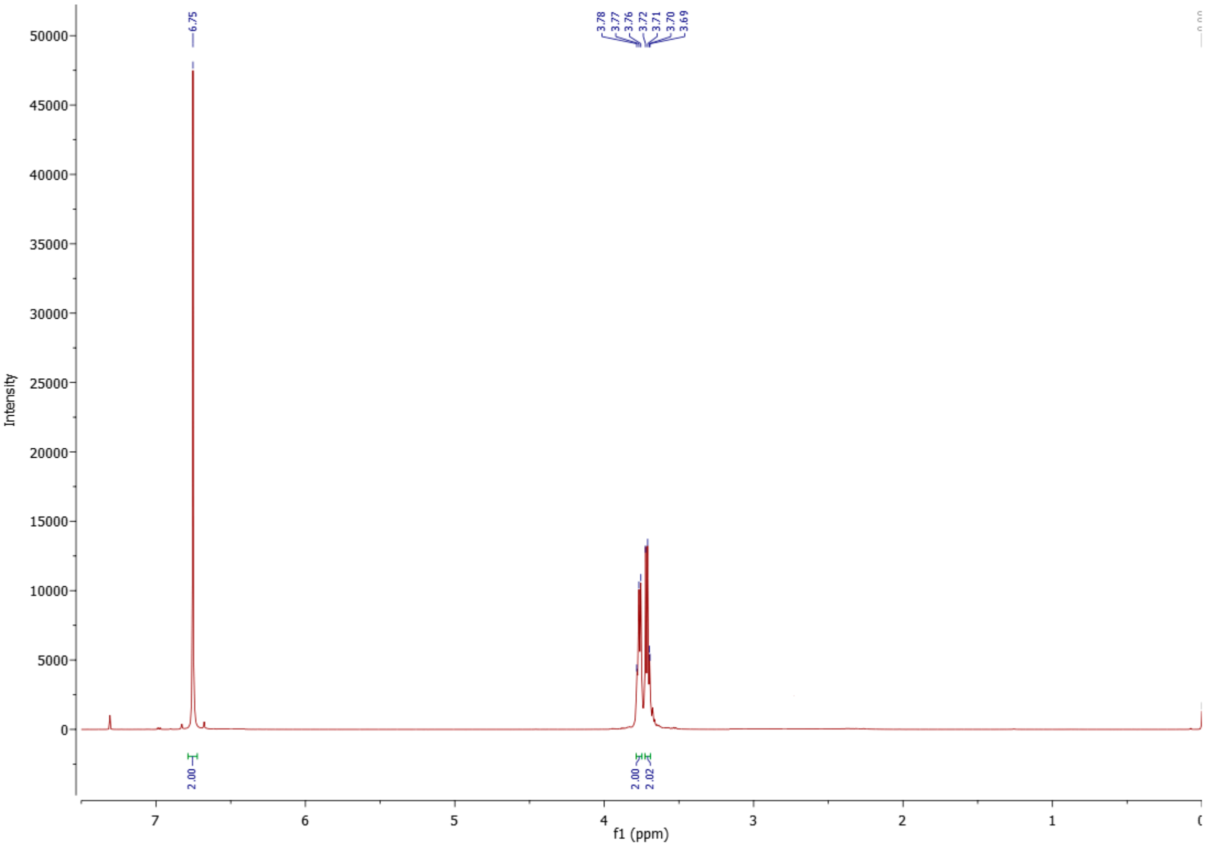

Figure S2. ^{1}H NMR HPD spectrum

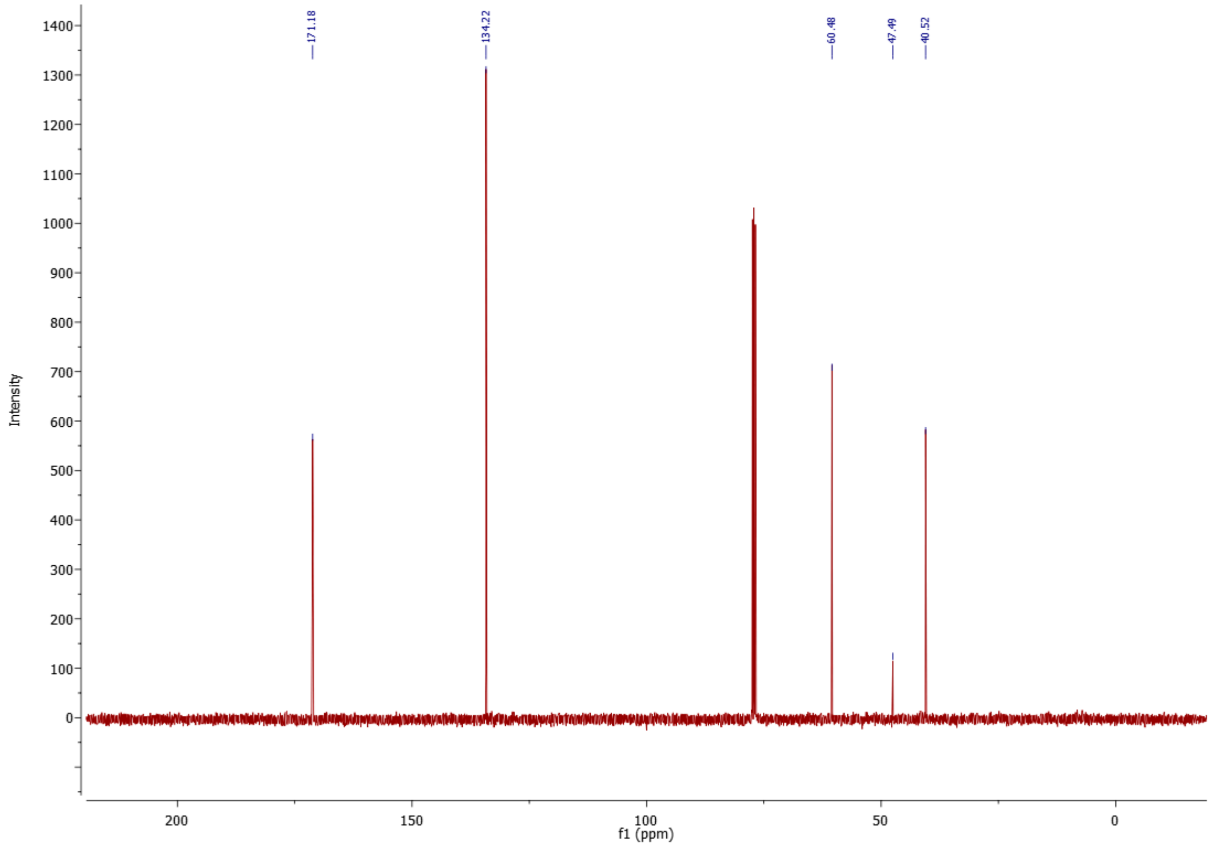

Figure S3. ^{13}C NMR HPD spectrum

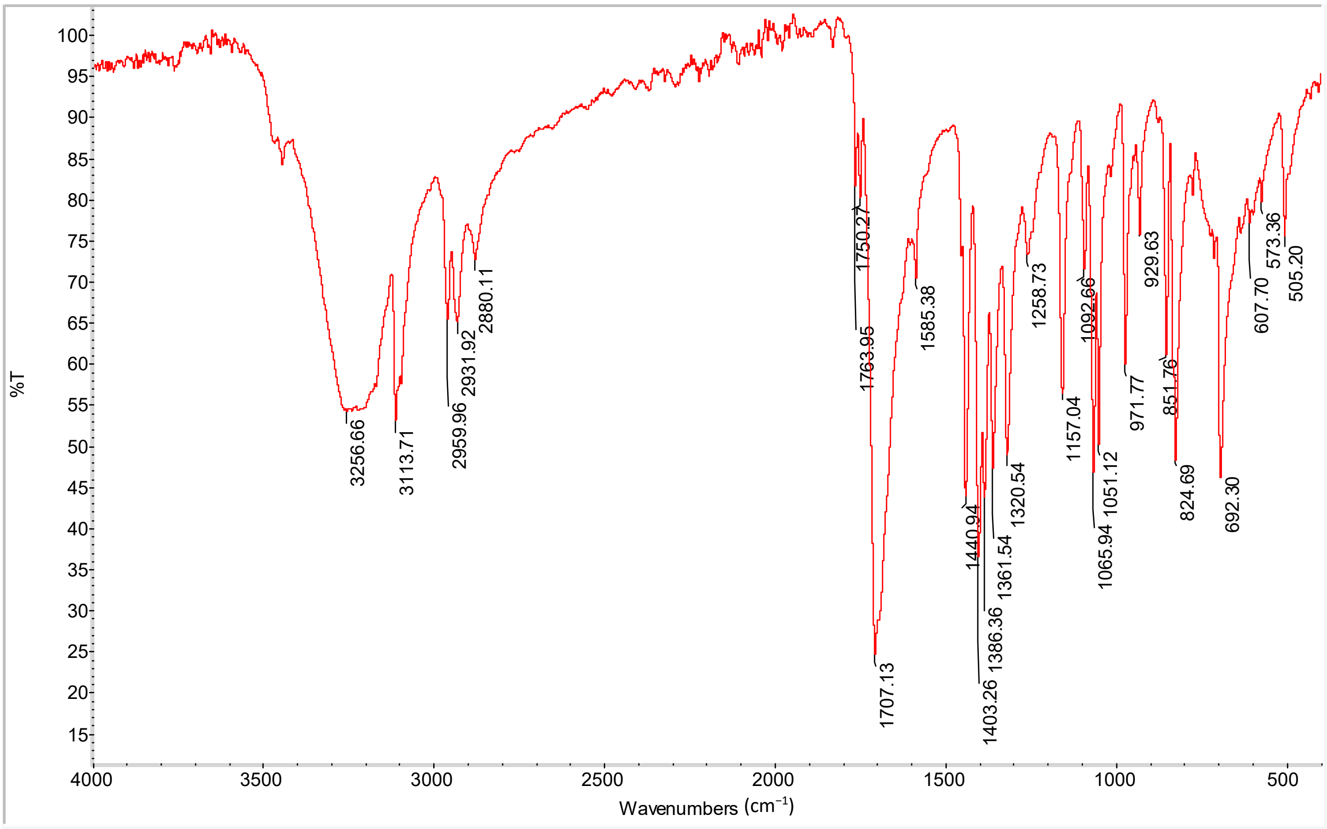

Figure S4. ATR-FTIR HPD spectrum

Step 4 – Synthesis of HODA (Hydroxymethyl)-10-oxatricyclo[5.2.1.02.6]dec-8-ene-3,5-dione-2-aminoethanol)

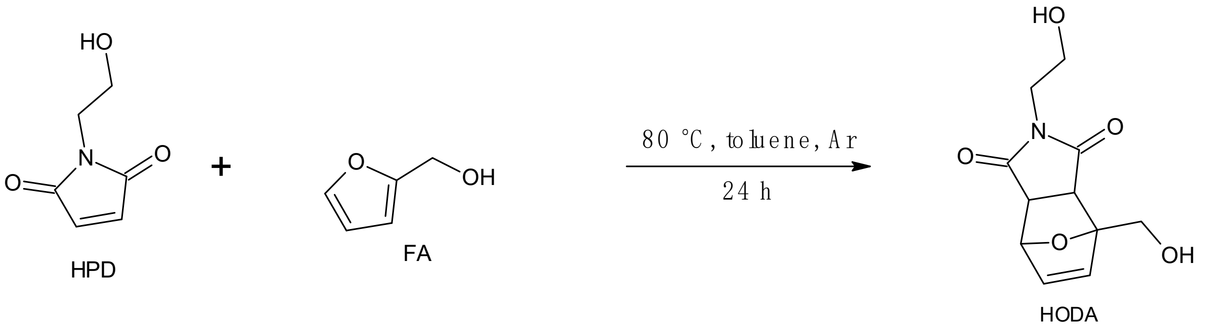

Figure S5. Scheme of the reaction of obtaining HODA

HODA (Hydroxymethyl)-10-oxatricyclo[5.2.1.02.6]dec-8-ene-3,5-dione-2-aminoethanol)

^{1}H NMR (400 MHz, $CDCl_3$, 298 K) δ (ppm) 6.61 (d, 1H), 6.55 (t, 1H), 5.28 (s, 1H), 4.11 (s, 2H), 3.76 (t, 2H) , 3.69 (t, 2H) , 3.04 (d, 1H), 3.01 (d, 1H), 2.85 (s, 1H), 2.28 (s, 1H); **^{13}C NMR** (400 MHz, $CDCl_3$, 298 K) δ (ppm): 138.31; 136.96; 91.50; 80.96; 60.72; 60.16; 49.99; 48.17; 41.75; **FT-IR (cm^{-1})** 417, 456, 499, 522, 584, 613, 644, 678, 716, 731, 787, 805, 836, 844, 876, 932, 953, 977, 1012, 1036, 1074, 1091, 1111, 1159, 1185, 1248, 1272, 1289, 1303, 1326, 1348, 1363 1402, 1428, 1681, 1766, 2883, 2917, 2929, 2950, 2978, 3016, 3085, 3428.

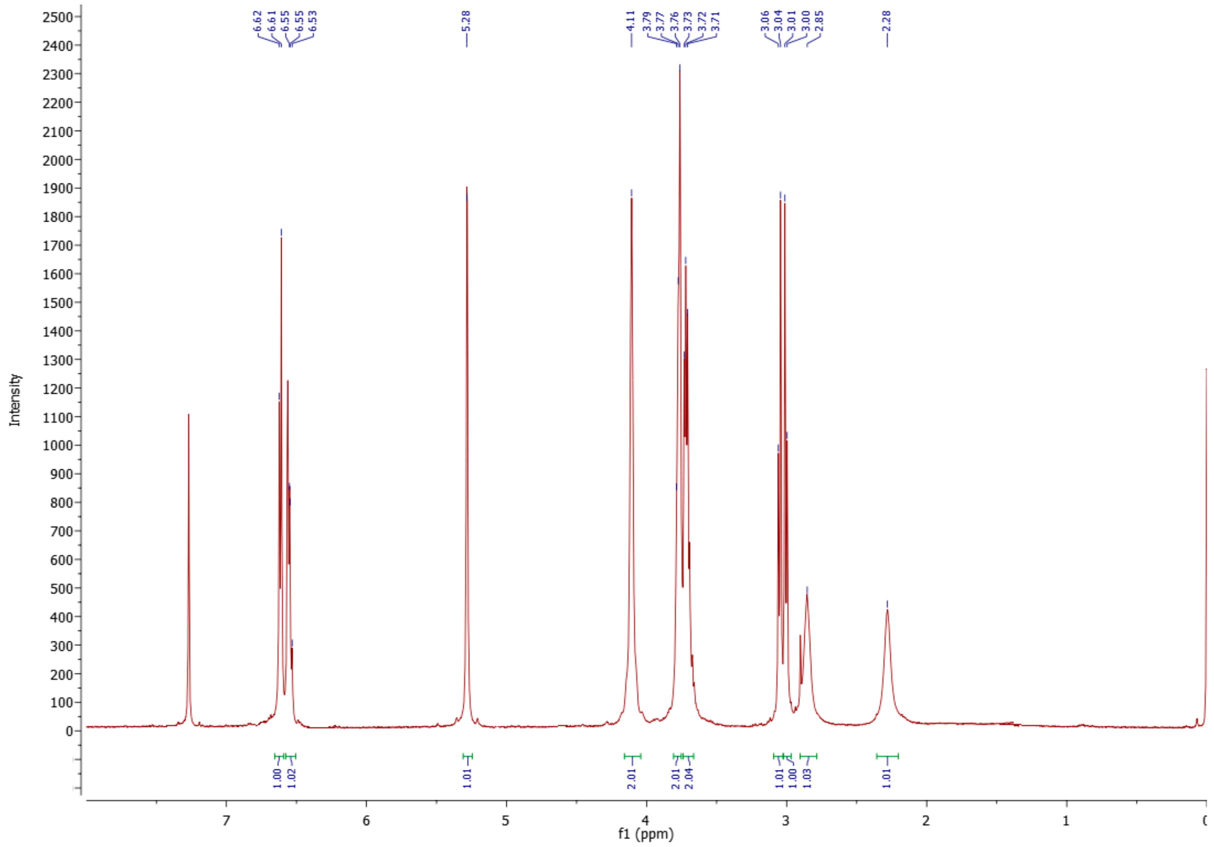

Figure S6. ^{1}H NMR HODA spectrum

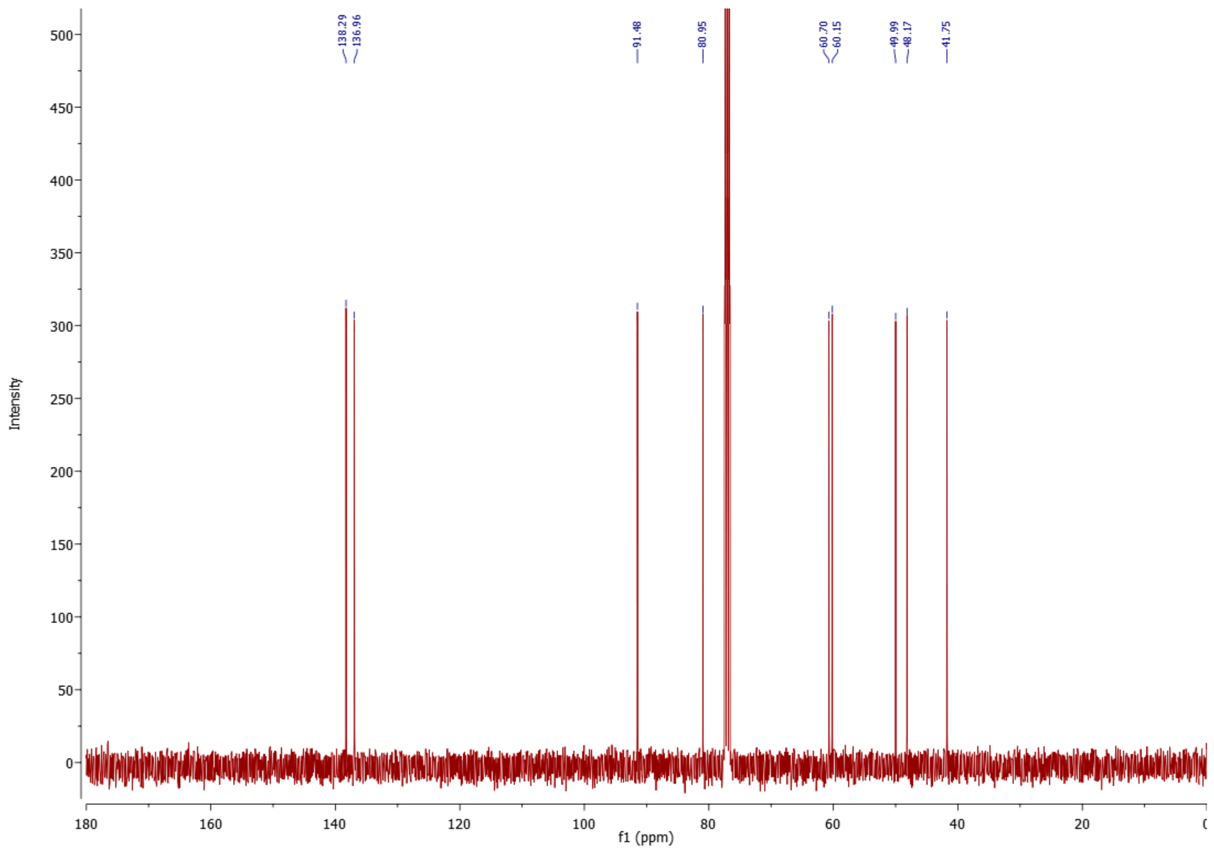

Figure S7. ^{13}C NMR HODA spectrum

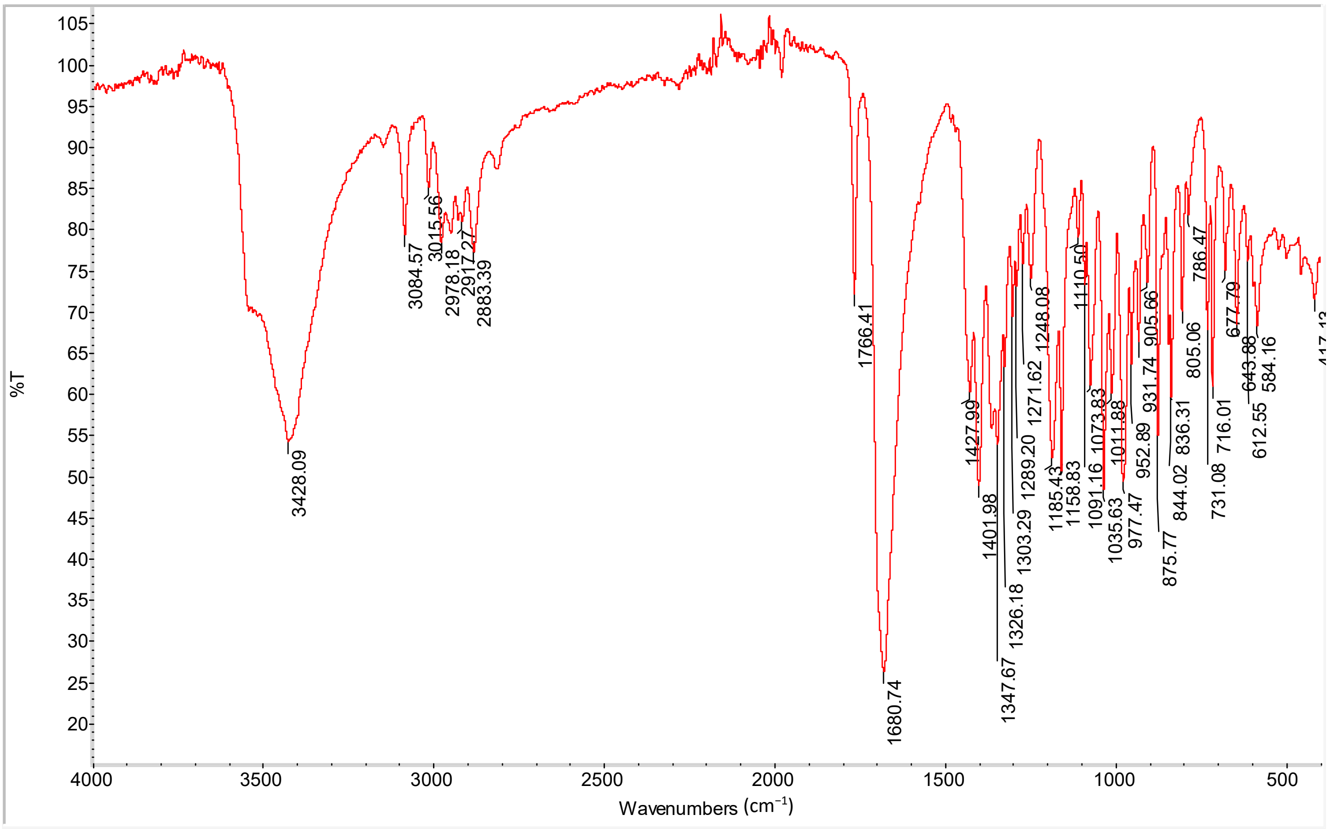

Figure S8. ATR-FTIR HODA spectrum

The FT-IR spectra during the reaction time for the obtained polyurethanes

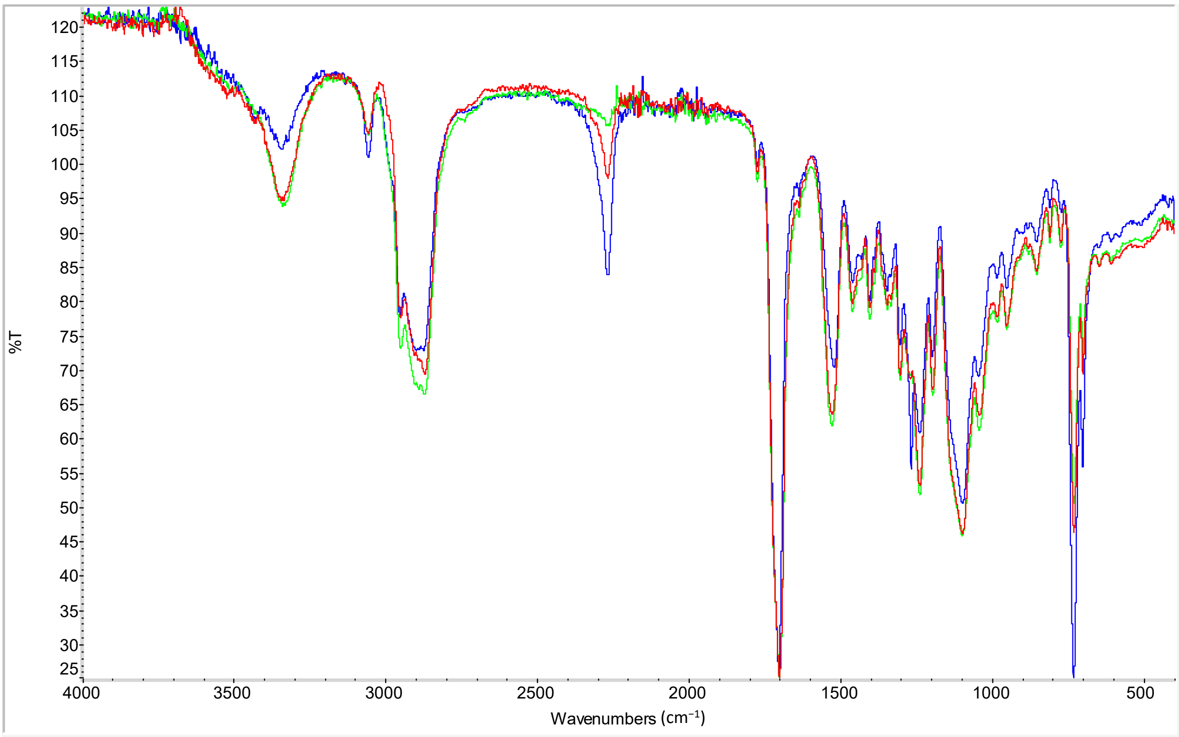

Figure S18. Comparison of FTIR spectra investigating the reaction of receiving UA-DA(HEA) after 0 (blue), 1 (red), and 3 h (green) of reaction time. Comparison of FTIR peaks: NCO stretching at 2270 cm^{-1}.

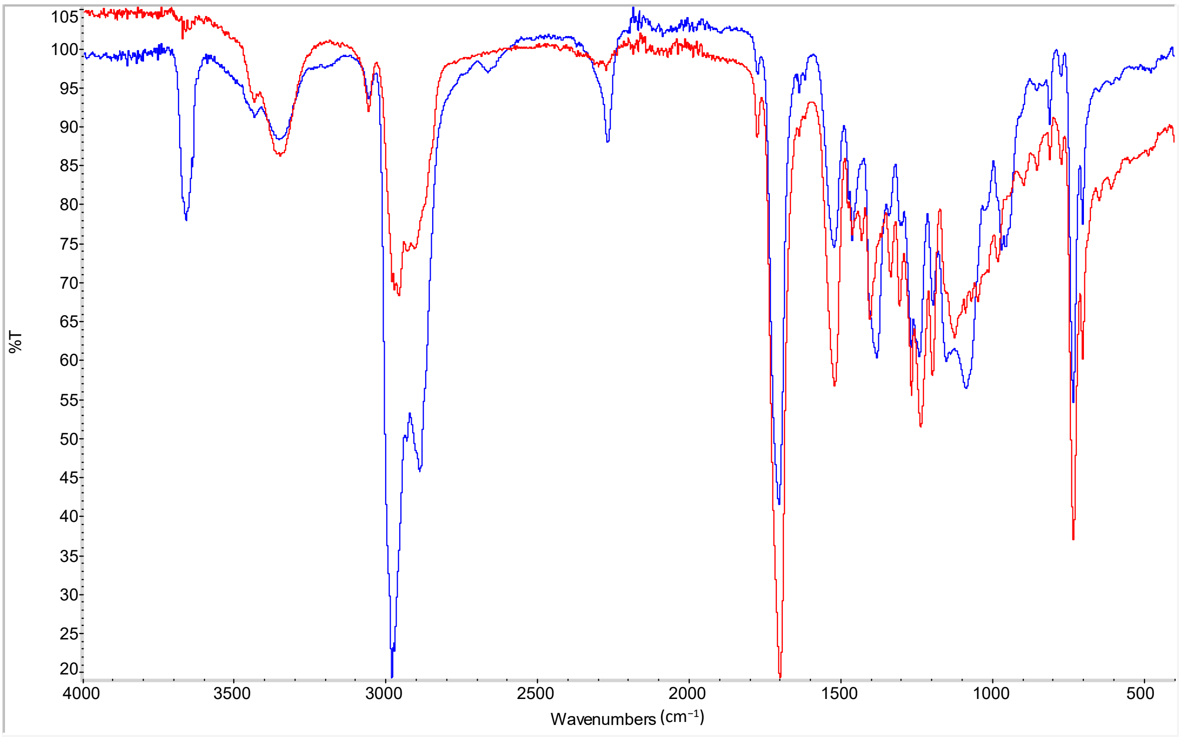

Figure S19. Comparison of FTIR spectra investigating the reaction of receiving UA-DA(HPA) after 0 (blue) and 1 h (red) of reaction time. Comparison of FTIR peaks: NCO stretching at 2270 cm^{-1}.

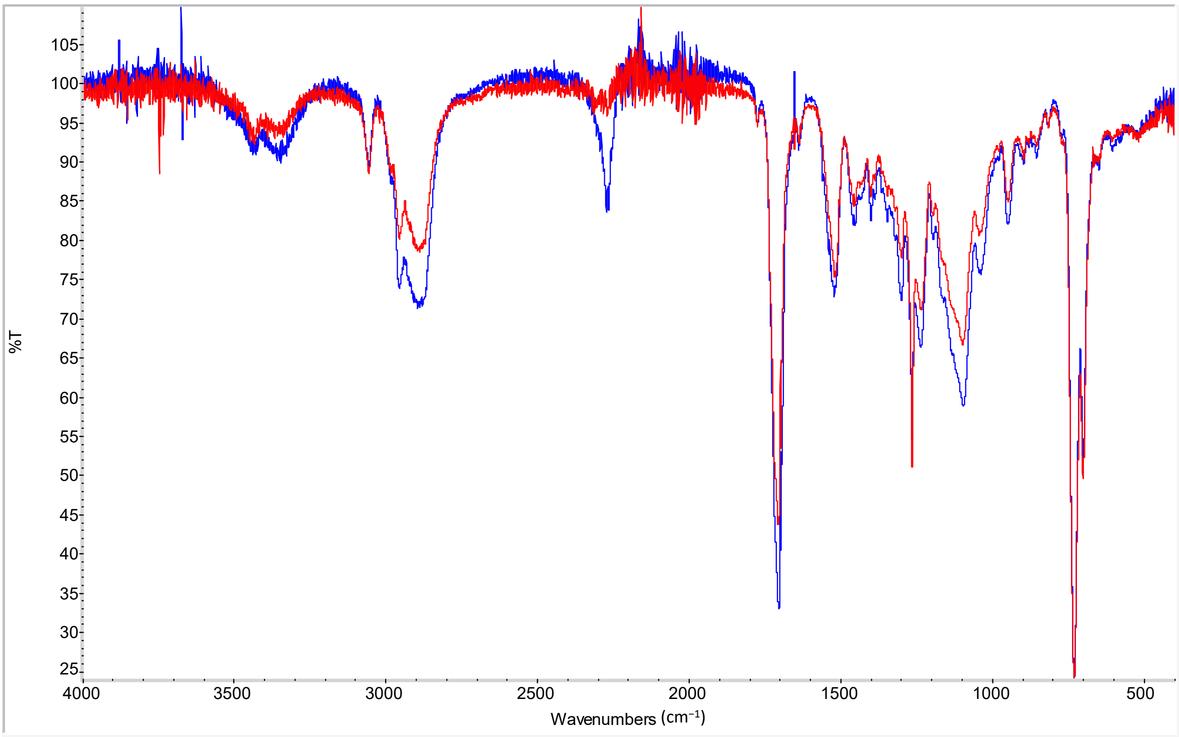

Figure S9. Comparison of FTIR spectra investigating the reaction of receiving UA-DA(HEMA) after 0 (blue) and 1 h (red) of reaction time. Comparison of FTIR peaks: NCO stretching at 2270 cm^{-1}.

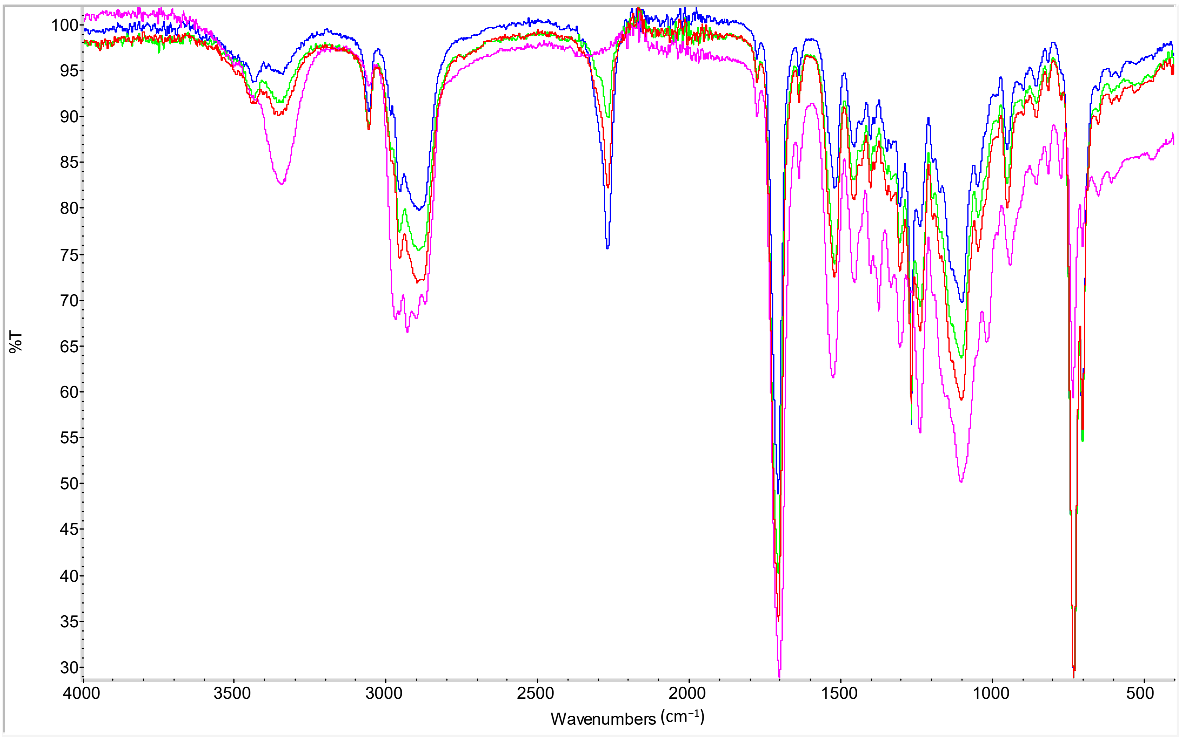

Figure S10. Comparison of FTIR spectra investigating the reaction of receiving UA-DA(HPMA) after 0 (blue), 1 (red), 4 (green), and 24 h (pink) of reaction time. Comparison of FTIR peaks: NCO stretching at 2270 cm^{-1}.

The NMR spectra of the obtained polyurethanes

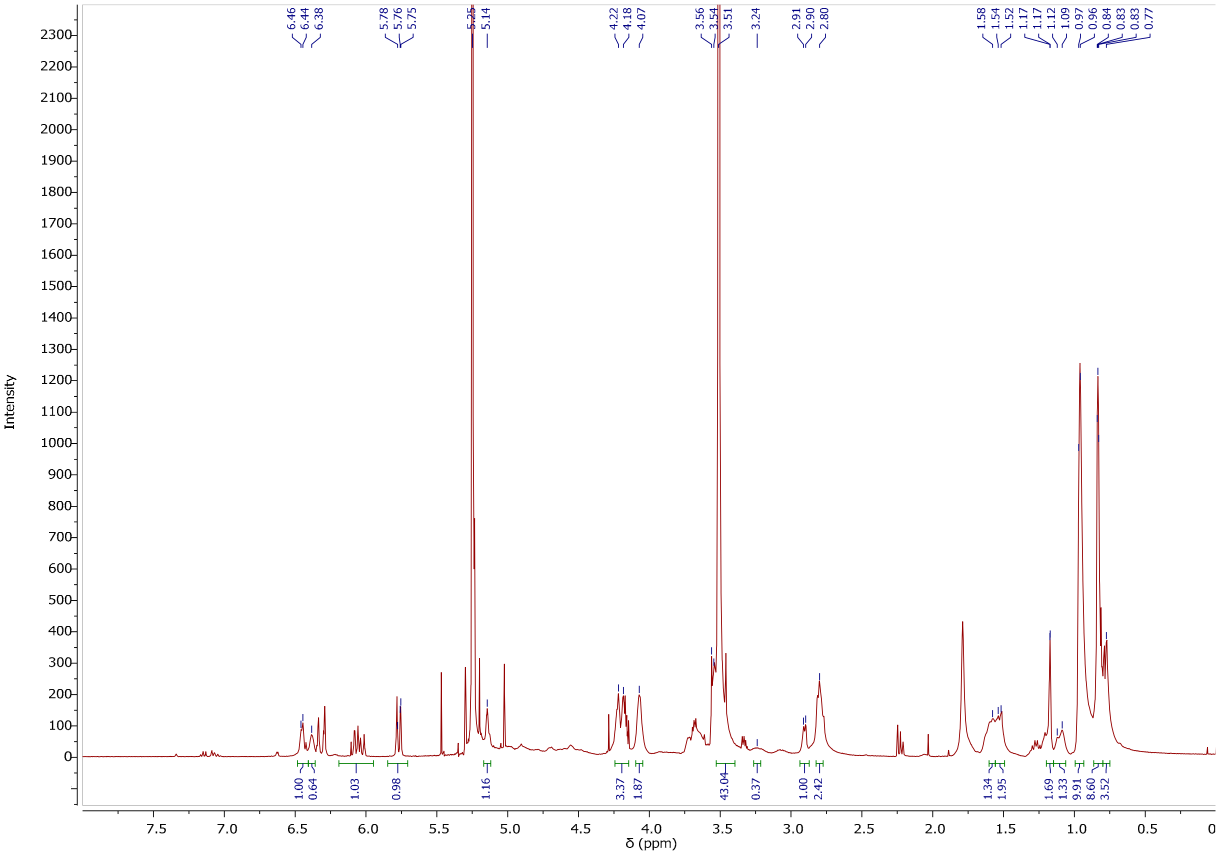

Figure S22. ^{1}H NMR spectra of UA-DA(HEA).

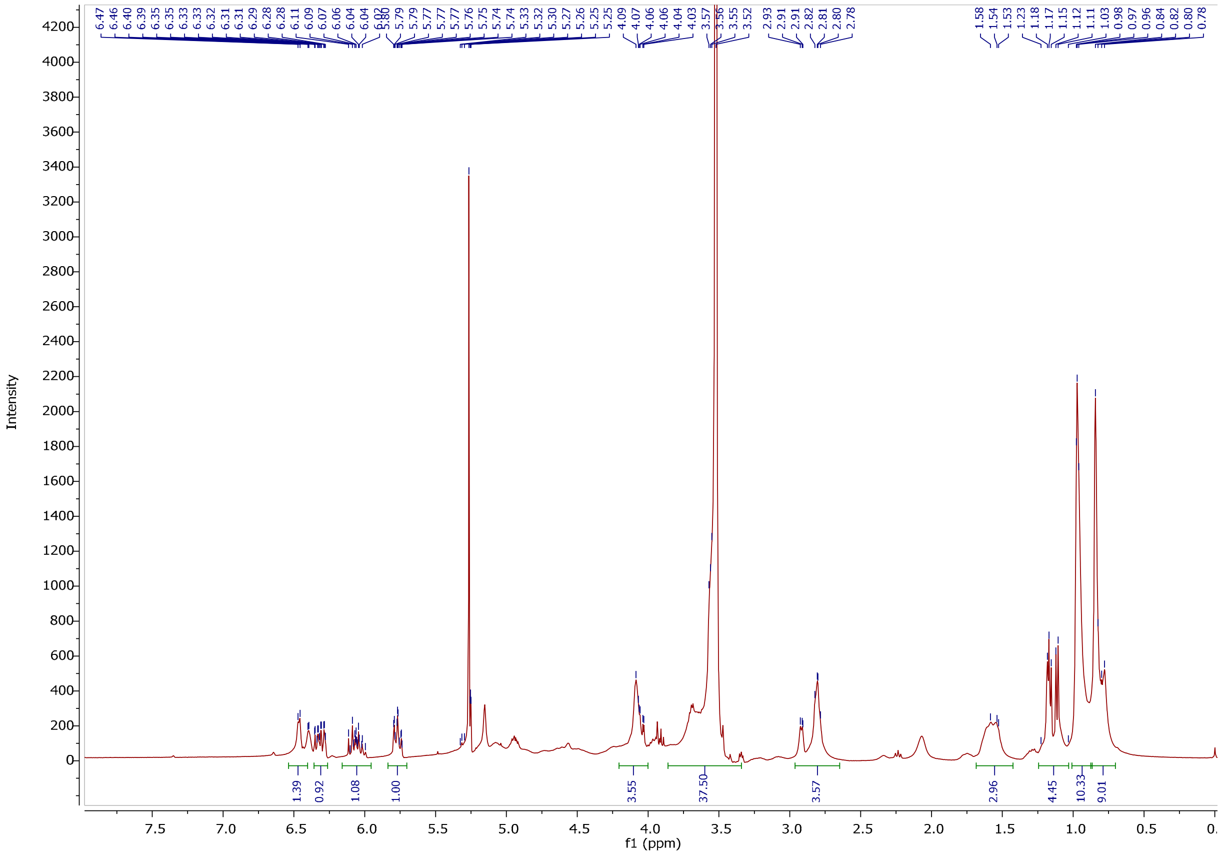

Figure S23. ^{1}H NMR spectra of UA-DA(HPA).

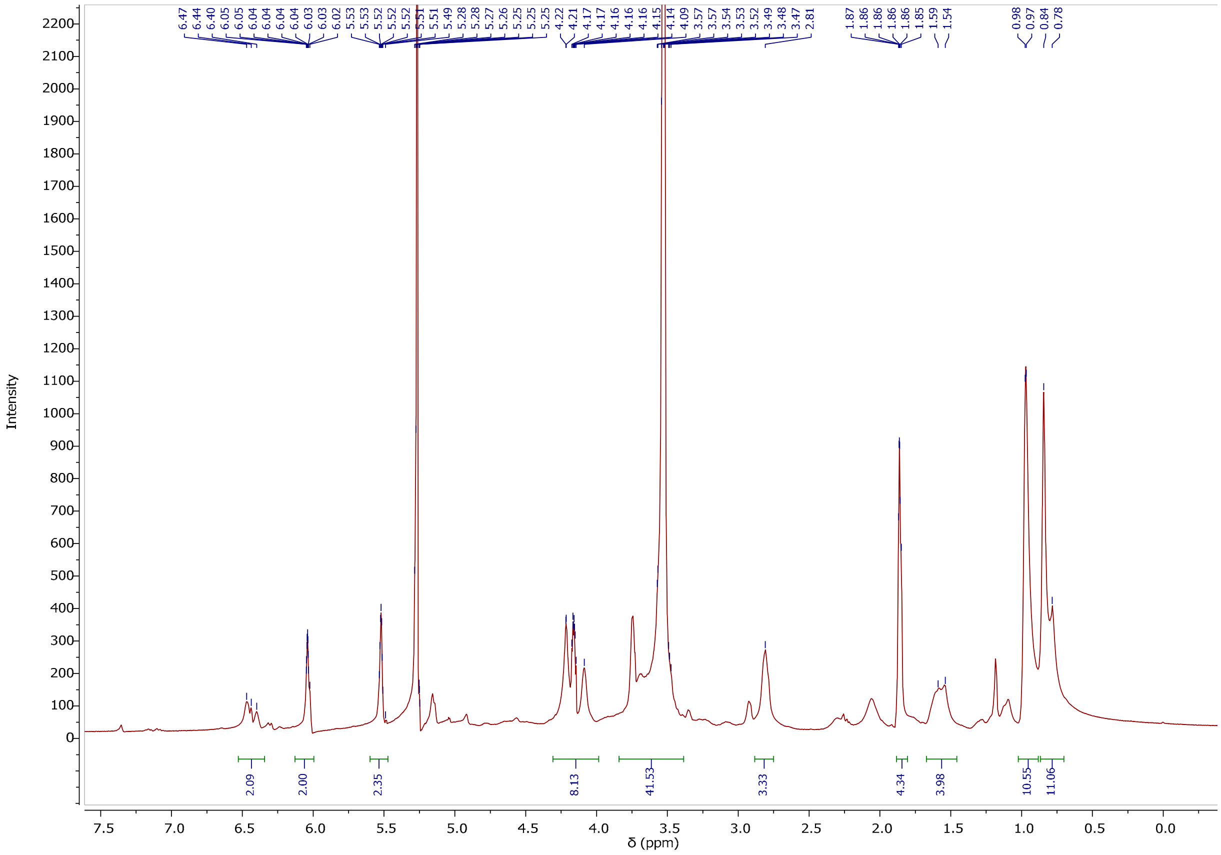

Figure S24. ^{1}H NMR spectra of UA-DA(HEMA).

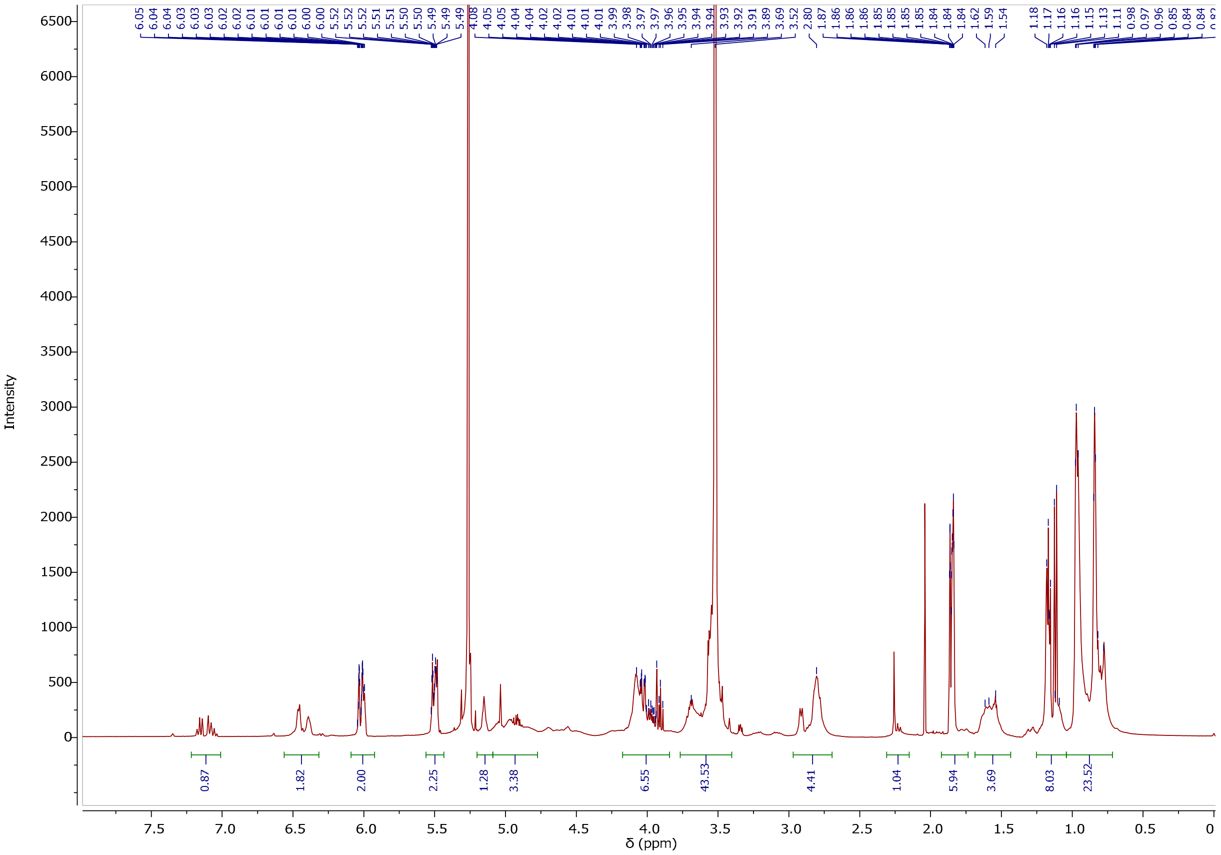

Figure S25. ^{1}H NMR spectra of UA-DA(HPMA).

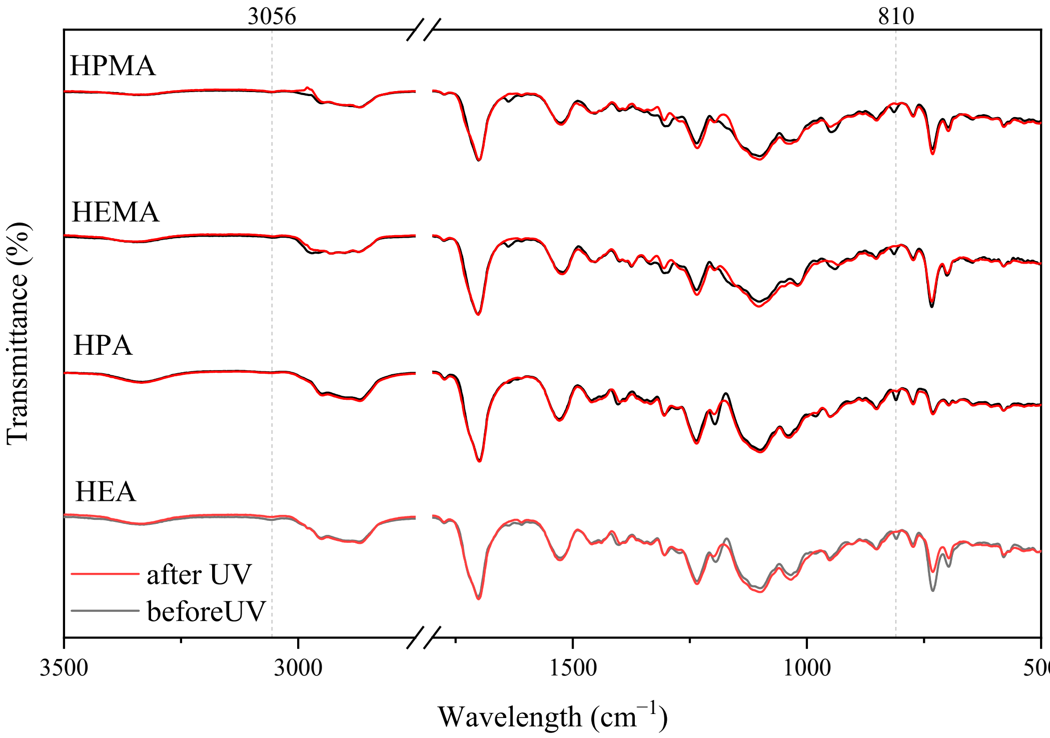

Figure S26. FTIR spectra of photoreactive compositions based on obtained resins, before and after UV irradiation.

The TG and DTG curves of the obtained urethane (meth)acrylates

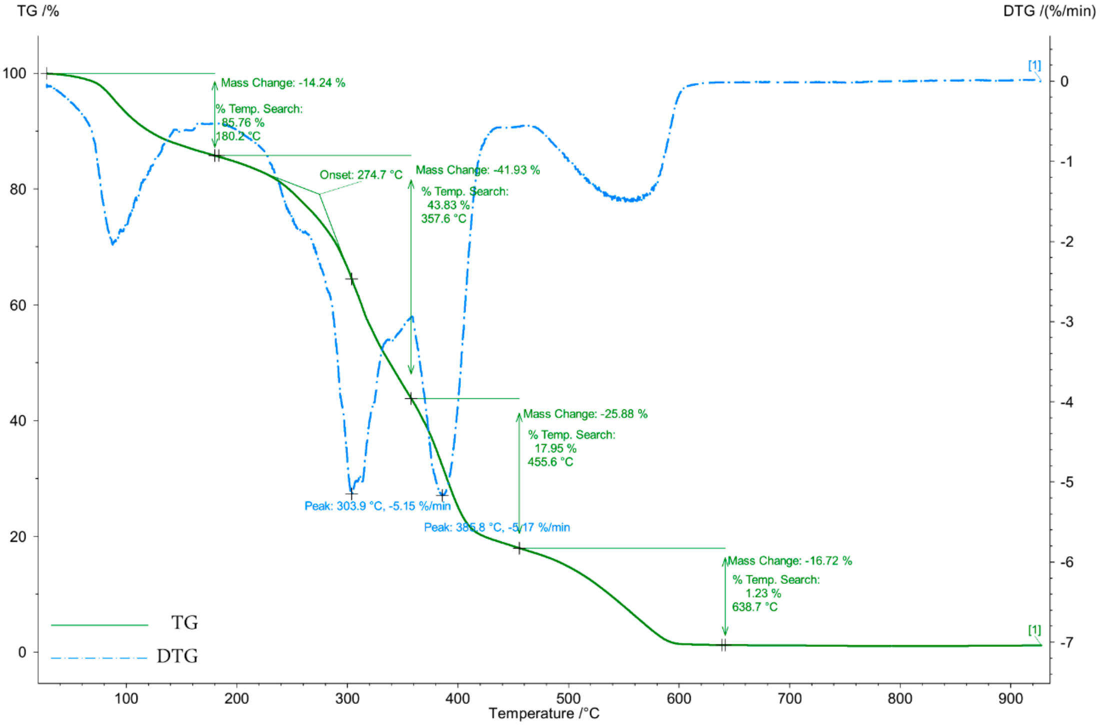

Figure S27. TG and DTG curves of UA-DA(HEA).

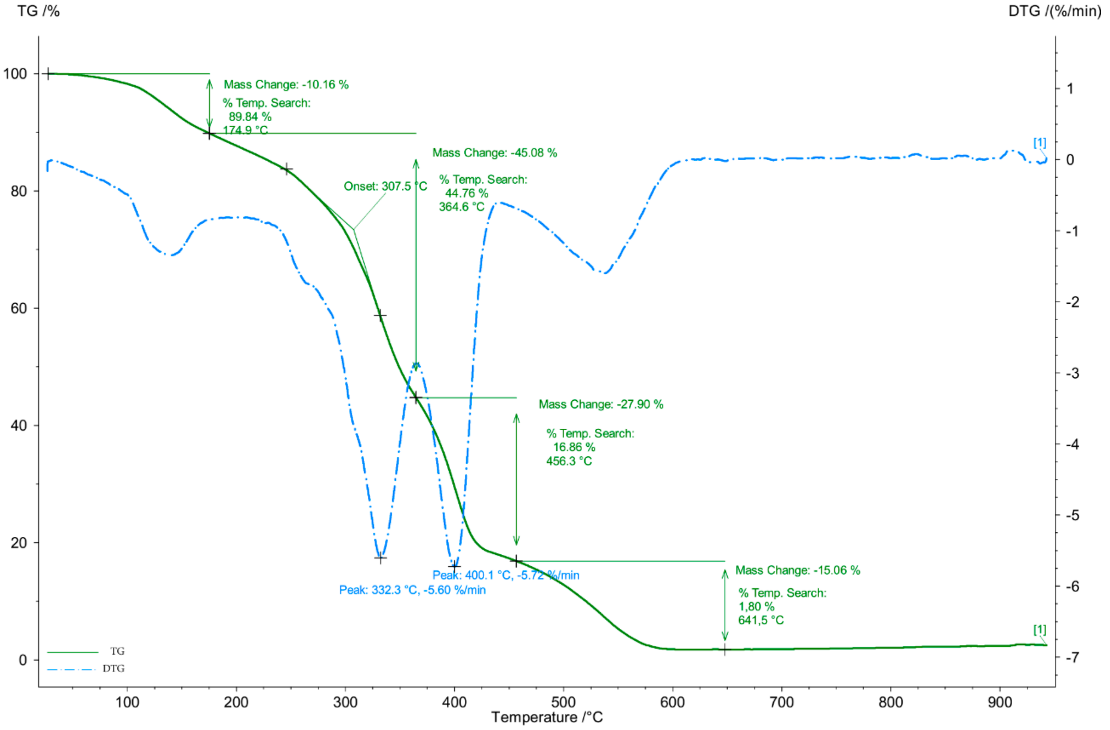

Figure S28. TG and DTG curves of UA-DA(HPA).

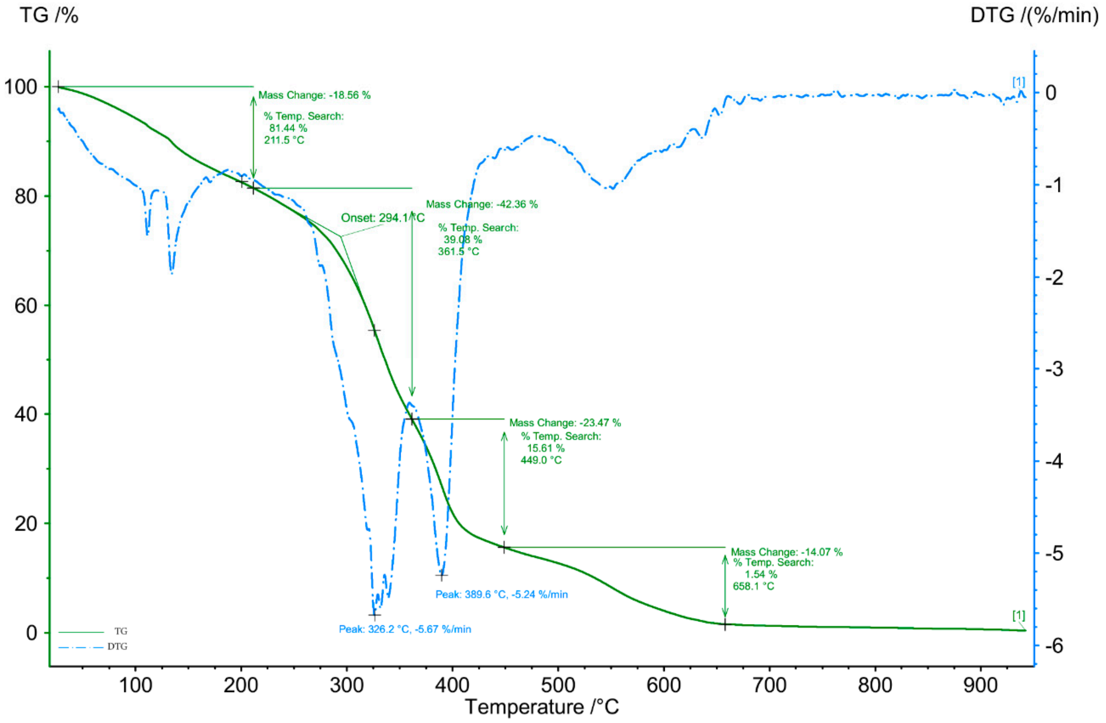

Figure S29. TG and DTG curves of UA-DA(HEMA)

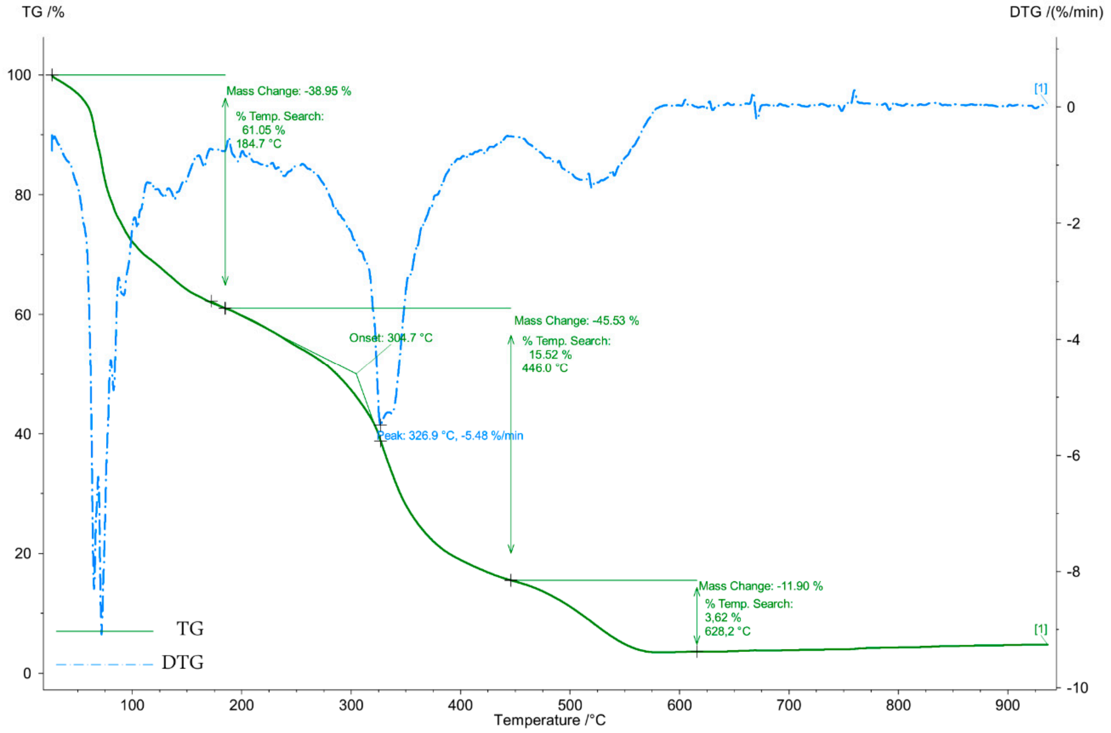

Figure S30. TG and DTG curves of UA-DA(HPMA)
